# Multifunctional scaffold inspired by hepatocyte exosomes promotes bone regeneration by regulating osteogenic differentiation via PI3K/AKT pathway

**DOI:** 10.1016/j.mtbio.2026.103360

**Published:** 2026-06-20

**Authors:** Yifan Zhang, Jie He, Yuyang Zeng, Yangyang Song, Zhengxing Wang, Qian Wang, Zhen You, Jiaming Sun

**Affiliations:** a Department of Plastic Surgery, Union Hospital, Tongji Medical College, Huazhong University of Science and Technology, Wuhan, 430022, China; b Department of Neurosurgery, The Union Hospital of Tongji College, Huazhong University of Science and Technology, Wuhan, 430022, China; c Department of Dermatology, Tongji Hospital, Tongji Medical College, Huazhong University of Science and Technology, Wuhan, 430030, China; d Medical Genetics Center, Maternal and Child Health Hospital of Hubei Province, Wuhan, Hubei Province, 430070, China; e Hubei Key Laboratory of Regenerative Medicine and Multi-disciplinary Translational Research (Huazhong University of Science and Technology), Wuhan, Hubei, 430022, China; f Department of Rehabilitation Medicine, Sichuan Provincial People's Hospital, School of Medicine, University of Electronic Science and Technology of China, Chengdu, Sichuan, 610065, China; g Rehabilitation Medicine Center, West China Hospital, Sichuan University, Chengdu, Sichuan, 610065, China; h Division of Biliary Surgery, Department of General Surgery, West China Hospital, Sichuan University, Chengdu, Sichuan, 610041, China; i Research Center for Biliary Diseases, West China Hospital, Sichuan University, Chengdu, Sichuan, 610041, China

**Keywords:** Liver-bone cross-talk, Bioprinting, Hepatocyte-derived exosomes, PI3K/AKT pathway, Osteogenesis

## Abstract

Exosome-mediated tissue-tissue communication represents a fundamental mechanism that maintains physiological homeostasis. This study proposes a novel therapeutic approach based on liver-bone cross-talk, wherein hepatocyte-derived exosomes (h-EXOs) markedly accelerated the repair of critical cranial defects. Specifically, h-EXOs were anchored onto a digital light processing (DLP)-printed scaffold (PH/PDA) composed of polycaprolactone macromolecule polymer (PCLMA) and nano-hydroxyapatite (nHap) via a polydopamine (PDA) coating to promote cranial bone regeneration. These PH/PDA scaffolds substantially enhanced bone mesenchymal stem cells (BMSCs) adhesion and proliferation, while the incorporated h-EXOs significantly promoted angiogenesis and osteogenic differentiation. Moreover, RNA sequencing revealed that h-EXOs were enriched in cargoes governing diverse cellular processes and activated the PI3K/AKT pathway to promote BMSCs osteogenesis. Following implantation, PH/PDA/h-EXOs scaffolds induced substantial defect closure and fostered a regenerative microenvironment similar to native calvarial tissue, characterized by an expansion of anti-inflammatory M2 macrophages and osteoblasts alongside pronounced vascularization. Overall, this study leverages inter-organ crosstalk in conjunction with personalized scaffold fabrication to propose a novel tissue-engineering strategy for enhancing tissue repair.

## Graphical abstract

**Figure:**
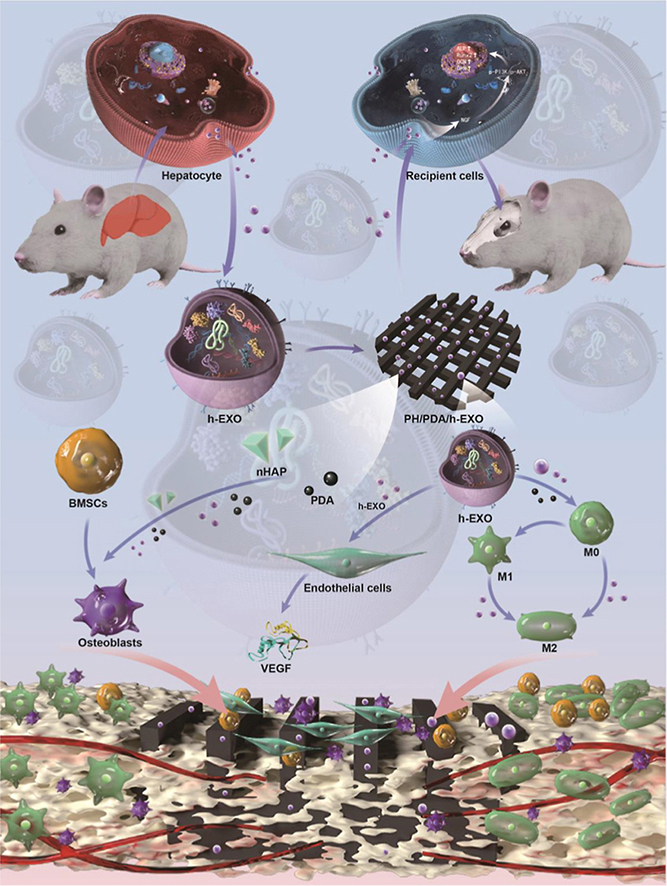

## 1 Introduction

**Scheme 1:**
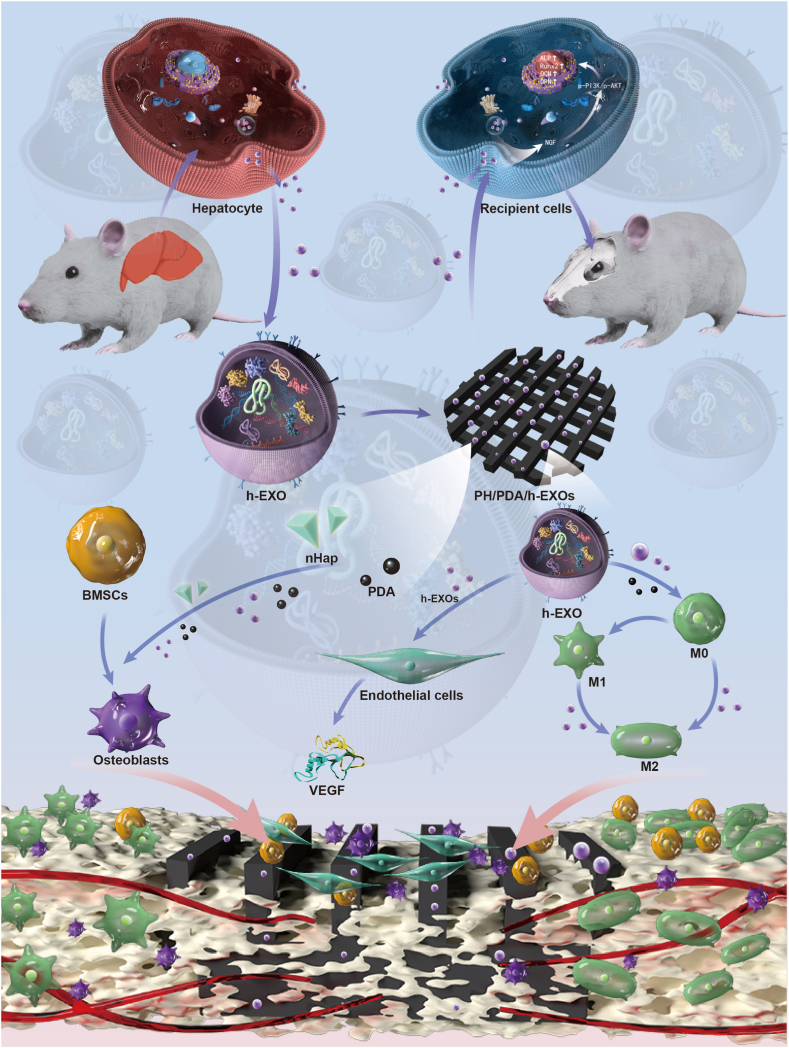
Schematic illustration of a cranial scaffold designed based on organ -organ cross-talk. Hepatocyte-derived exosomes are delivered via the scaffold to the cranial defect site, where they are internalized by recipient cells, subsequently activating the PI3K/AKT signaling pathway to promote cranial bone regeneration. The PH/PDA/h-EXOs composite scaffold consists of a PCLMA-nHap scaffold, a PDA coating, and loaded h-EXOs.

In the complex physiological regulatory network of the human body, various organ systems coordinate to maintain homeostasis through hormone signaling, metabolic products, and neural regulation [[1], [2], [3]]. Interactions between different organs play a crucial role in this process [[4], [5], [6]]. For instance, coagulation factor XI (FXI) expressed in the liver activates the bone morphogenetic protein (BMP)–SMAD1/5 pathway in cardiac tissue, thereby regulating cardiac gene expression and improving diastolic function [7]. Meanwhile, the spleen promotes cardiac remodeling through CD23-mediated signaling pathways [8]. These tissue-specific axes collectively modulate systemic metabolic homeostasis and promote tissue repair under pathological conditions (see Scheme 1).

As is well documented, bone tissue is an indispensable component of the human body [9]. However, large bone defects induced by severe trauma or malignancies exceed the regenerative capacity, resulting in loss of related functions and a significant decrease in the patient's quality of life [10,11]. Bone repair involves the coordinated actions of various cell types, including osteoblasts, vascular endothelial cells, and immune cells, to repair the defective area [12,13]. While autologous bone grafting remains the gold standard for clinical reconstruction, the availability of autologous bone is limited [14]. Synthetic bone substitutes are challenging to individualize and fail to precisely replicate the geometry of the defective area [15]. Although the introduction of stem cells can improve repair efficiency, several limitations, including immune rejection and low survival rates, cannot be overlooked [16,17].

The bone and liver are functionally interdependent organs [1,18,19]. As a key organ, bone plays a decisive role in protecting visceral metabolic organs and participating in human metabolism [20,21]. Similarly, the liver serves as a central metabolic hub that regulates the metabolism of glucose, protein, and vitamin D [22,23]. Earlier studies have identified various signaling axes between bone tissue and the liver that regulate their respective functions. Bone tissue affects the physiological functions of other organs by regulating glucose metabolism through the secretion of osteocalcin, lipocalin-2, and other bone-derived factors [19,24,25]. Vitamin D is converted into 25-hydroxyvitamin D in the liver by 25-hydroxylase, and changes in this metabolic pathway have been associated with the development of osteoporosis [2]. These studies conjointly indicate that the liver-bone communication pathway is crucial for preserving the stability and development of organs. However, studies comprehensively exploring pathways through which hepatic function contributes to bone defect repair are scarce.

Notably, exosomes (EXO) are synthesized by various cell types and contain numerous active substances, encompassing RNAs, proteins, and lipids [26,27]. They have a diameter ranging from 20 to 140 nm and are released via vesicular secretion [28]. In addition, they fuse with target cells and deliver their cargo, thereby altering cellular differentiation and function [29]. Mounting evidence indicates that EXOs play a critical role in the transport and exchange of substances across cells, organs, and tissues, where they exert pleiotropic effects [26,30,31].

In recent years, the application of three-dimensional (3D) printed scaffolds to promote bone regeneration has garnered extensive attention owing to their versatility and excellent performance [[32], [33], [34]]. Among them, DLP 3D printing has rapidly developed due to its high printing accuracy and fabrication speed [35]. By selecting hydrogels or resins with photosensitive properties, layer-by-layer curing of the hydrogel and resincan be achieved under a digital model to manufacture high-precision biomimetic scaffolds [36,37].

The present study proposed a method for the treatment of bone defects based on inter-organ interactions. EXOs were initially extracted from hepatocytes, and their efficient internalization by BMSCs was confirmed. Next, a high-precision cranial scaffold termed PH/PDA was fabricated by combining PCLMA with nHap and coating the surface with a PDA layer. Subsequently, h-EXOs were loaded onto the PH/PDA scaffold. In vitro experiments demonstrated that the scaffold containing h-EXOs significantly promoted angiogenesis and osteogenesis. Meanwhile, RNA sequencing was performed on BMSCs cultured with h-EXOs to investigate the effects of h-EXOs on osteogenesis. Finally, the efficacy of the scaffold was evaluated using a rat cranial defect model, and the results indicated that the h-EXOs-loaded PH scaffold significantly promoted bone repair at 4 and 12 weeks following implantation.

## 2 Results

### 2.1 Characterization of EXOs secreted from hepatocytes

**Fig. 1:**
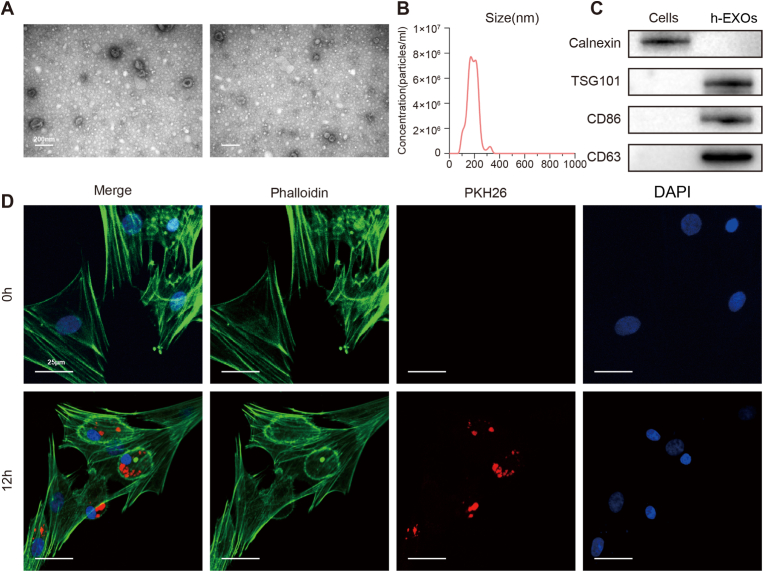
**Characterization and internalization of h-EXOs. A.** The morphology of exosomes under TEM. **B.** Particle size distribution of h-EXOs by NTA. **C.** WB analysis of the exosome-specific markers (CD63, CD81, TSG101) and the endoplasmic reticulum marker Calnexin. **D.** The uptake of h-EXOs by BMSCs. FITC-phalloidin (green) and DAPI (blue) were used to stain the cytoskeleton and nucleus of BMSCs, respectively, while the h-EXOs were labeled with Dil (red). (For interpretation of the references to color in this figure legend, the reader is referred to the Web version of this article.)

To begin, primary hepatocytes with binucleated structures were successfully isolated (Fig. S1A, Supporting Information). Subsequently, the exoEasy Maxi Kit was employed to extract EXOs from hepatocytes. High-magnification transmission electron microscopy (TEM) images depicted that h-EXOs were spherical particles with dimensions ranging approximately from 100 to 250 nm (Fig. 1A and B). Western blot (WB) analysis suggested that the isolated h-EXOs contained the specific markers CD63, CD81, and TSG101, whereas the endoplasmic reticulum marker calnexin was nearly undetectable (Fig. 1C). These results indicated that h-EXOs were successfully isolated from the culture medium. Subsequently, the internalization capacity of h-EXOs by BMSCs was examined. As illustrated in Fig. 1D, after 12 h of co-culture with Dil-labeled h-EXOs, red fluorescence was clearly visible in the cytoplasm of BMSCs, most prominently surrounding the nucleus.

Thereafter, h-EXOs were subjected to RNA sequencing to identify their cargo (Fig. S2A, Supporting Information). The encapsulated cargo was associated with several biological processes, including osteoblast differentiation, angiogenesis, and immune regulation, as demonstrated by Kyoto Encyclopedia of Genes and Genomes (KEGG) and Gene Ontology (GO) analyses (Fig. S2B and S2C, Supporting Information). Moreover, protein-protein interaction network analysis displayed that these interactions are implicated in energy metabolism, signal transduction, and cell-cycle regulation (Fig. S2D and S2E, Supporting Information).

### 2.2 Biological effects of hepatocyte-derived EXOs in vitro

**Fig. 2:**
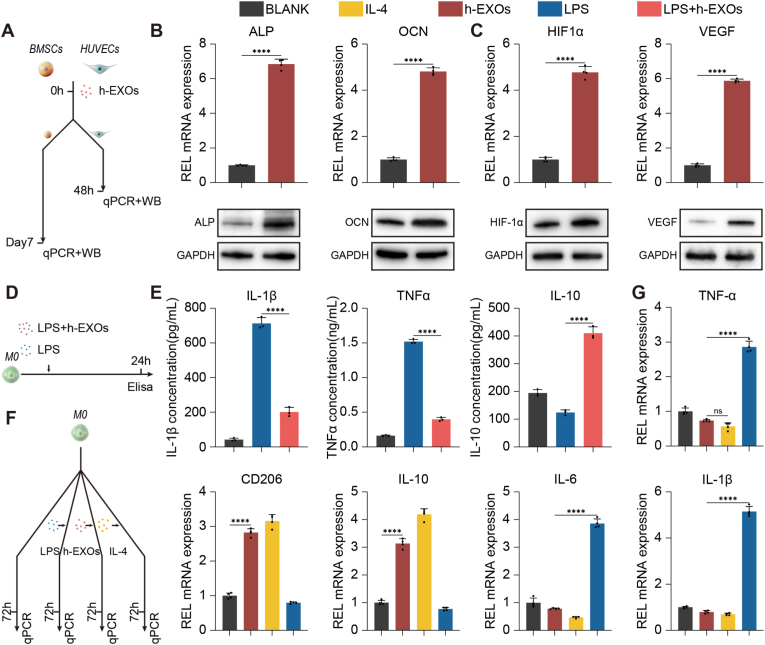
**The biological effects of h-EXOs in vitro. A.** Schematic diagram of BMSCs and HUVECs co-cultured with h-EXOs. **B.** In BMSCs co-cultured with h-EXOs, the mRNA and protein expression levels of ALP and OCN were upregulated. (n = 4) **C.** In HUVECs co-cultured with h-EXOs, the mRNA and protein expression levels of ALP and OCN were upregulated. (n = 4) **D.** Schematic diagram of M0 macrophages treated with LPS and LPS + h-EXOs. **E.** Concentrations of IL-1β, TNFα, and IL-10 in the supernatant detected by ELISA. (n = 3) **F.** Schematic diagram of M0 macrophages treated with LPS, h-EXOs and IL-4. G. H-EXOs co-culture significantly downregulated upregulated M1 markers (TNF-α, IL-6, IL-1β) and upregulated M2 markers (CD206, IL-10) at the mRNA level in macrophages. (n = 4) ∗∗∗∗p < 0.0001.

Given their bioactivity, EXOs have garnered widespread attention in the field of tissue engineering. Previous studies have reported that they can modulate cell-to-cell communication and can be internalized by recipient cells to foster a microenvironment conducive to bone regeneration [30,31]. Thus, a co-culture system involving BMSCs, human umbilical vein endothelial cells (HUVECs) and h-EXOs were established in this study (Fig. 2A). The results demonstrated a significant increase in the gene expression level of alkaline phosphatase (ALP) and osteocalcin (OCN) at 7 days post-induction (Fig. 2B). Angiogenesis is a critical component of bone formation. Therefore, the expression levels of angiogenesis-related genes, including hypoxia-inducible factor 1-alpha (HIF-1α) and vascular endothelial growth factor (VEGF), were assessed after 48 h of co-culture using quantitative real-time polymerase chain reaction (qRT-PCR) and WB (Fig. 2C). As anticipated, the results signaled that h-EXOs enhanced both osteogenic differentiation and angiogenesis.

Furthermore, the present study explored the regulatory effects of h-EXOs on the polarization of RAW264.7 macrophages toward either the pro-inflammatory M1 or anti-inflammatory M2 phenotype (Fig. 2D) [38]. Following a 24-h induction period, the levels of pro-inflammatory cytokines associated with M1 polarization, including interleukin-1β (IL-1β) and tumor necrosis factor-α (TNF-α), were markedly lower in the LPS + h-EXOs scaffold group compared to the LPS-only group (Fig. 2E). To further elucidate the phenotypic shift, qRT-PCR analysis was conducted on day 3 to assess the expression of phenotype-specific genes (Fig. 2F). The expression levels of M1-related genes, namely TNF-α, IL-6, and IL-1β, were significantly lower in both the h-EXOs and IL-4 treatment groups compared to the Blank and LPS controls. In contrast, the transcriptional levels of M2-associated markers, such as CD206 and IL-10, were significantly upregulated following h-EXOs or IL-4 stimulation (Fig. 2G). Taken together, these findings suggest that h-EXOs exert immunomodulatory effects by promoting macrophage polarization toward an anti-inflammatory M2-like phenotype, thereby contributing to the remodeling of the local immune microenvironment.

### 2.3 Printing and characterization of PCLMA/nHap

**Fig. 3:**
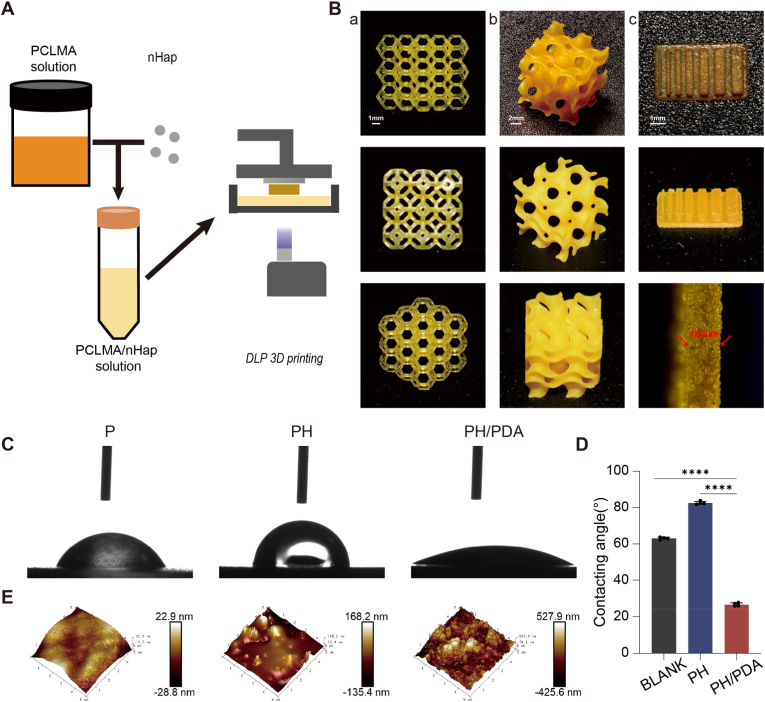
**Printing and characterization of PCLMA/nHap. A.** Schematic Illustration of DLP Printing of PCLMA/nHap. **B.** PCLMA/nHap can be printed into various high-precision models. **C.** Water contact angles of scaffolds. **D.** Quantitative analysis of water contact angle. (n = 4) **E.** The AFM of scaffolds. ∗∗∗∗p < 0.0001.

The printing accuracy of PCLMA/nHap (named PH) was evaluated by fabricating different structures and capturing images from multiple angles (Fig. 3A). The findings demonstrated that the PH system could fabricate diverse 3D architectures with high accuracy (Fig. 3B). Noteworthily, as indicated by the red arrow, it achieved a minimum feature size of approximately 100 μm (Fig. 3Bc). The surface of PCLMA exhibited strong biological inertness that can hinder bone integration at the interface [39]. Therefore, PDA was introduced [40,41]. Water contact angle measurements suggested that the PDA coating substantially decreased the hydrophobicity of the material surface (Fig. 3C and D). Additionally, atomic force microscopy (AFM) delineated that the PDA coating increased the surface roughness of the material (Fig. 3E). Collectively, these modifications are favorable for cellular adhesion.

### 2.4 Characterization and biocompatibility of PH/PDA scaffold

**Fig. 4:**
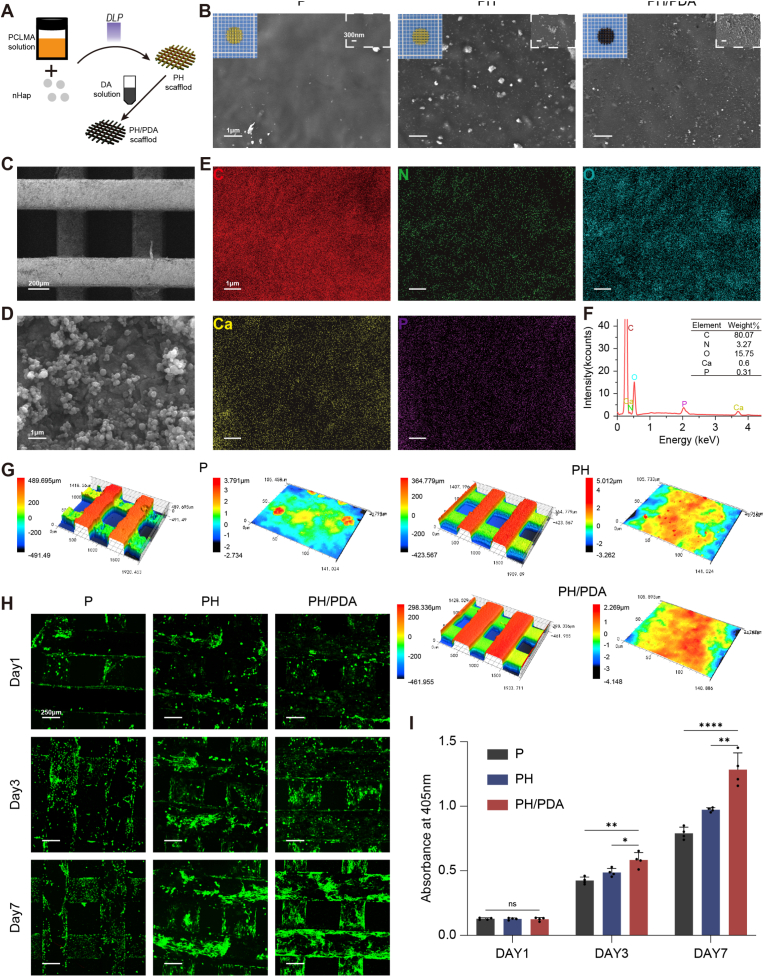
**Characterization and biocompatibility of PH/PDA scaffold. A.** Schematic illustration of PH/PDA scaffold fabrication. **B.** High-magnification SEM images and macroscopic views of P, PH, and PH/PDA scaffold surfaces. **C-D.** Low-magnification and high-magnification SEM image of the PH/PDA/h-EXOs scaffold. **E-F.** The EDS analysis confirmed the distribution of C, N, O, Ca and P in the scaffold. **G.** Surface topography and 3D micrographs of P, PH, and PH/PDA scaffold. **H-I.** Calcein-AM/PI staining and CCK-8 assay of BMSCs seeded on scaffolds at days 1, 3, and 7. (n = 4) ∗p < 0.05, ∗∗p < 0.01, ∗∗∗∗p < 0.0001.

High-precision PH/PDA scaffolds were constructed using a DLP 3D printer and dopamine solution (Fig. 4A). Both macroscopic and scanning electron microscopy (SEM) analyses revealed that the dimensions of the three scaffolds were identical, while the PH scaffold treated with PDA exhibited a black color (Fig. 4B). EDS mapping analysis confirmed a uniform distribution of C, N, O, P, and Ca throughout the scaffolds, confirming the successful incorporation of nHap and the even deposition of a PDA coating on the scaffold surface (Fig. S3, Supporting Information). EDS-spectrum tests indicated that the Ca/P weight ratio (wt%) in the PH and PH/PDA scaffolds was approximately 1.9, aligning with the standard nHap value reported in the literature. The results of electron microscopy indicated that the loading of exosomes did not alter the morphology of the scaffold and enabled uniform distribution on the scaffold surface (Fig. 4C and D). In contrast, in the absence of a PDA coating on the surface, exosomes were difficult to load onto the PH scaffold surface (Fig. S4, Supporting Information). Moreover, an increase in the N element weight detected by EDS-mapping further confirmed the successful loading of exosomes onto the scaffold (Fig. 4E and F).

Furthermore, the surface topography of the scaffolds was evaluated using a 3D laser scanning microscope, and 3D images were generated to illustrate the changes in surface morphology. In the presence of a laser, the scaffolds displayed a distinct color distribution, which may be attributed to the incorporation of nHap and PDA particles (Fig. 4G). The roughness analysis involved the measurement of two parameters: average roughness (Sa) and root mean square roughness (Sq). At the micron scale, the Sa values of the P, PH, and PH/PDA scaffolds were 0.603 μm, 0.852 μm, and 0.773 μm, respectively. Meanwhile, the Sq of P, PH, and PH/PDA scaffolds were 0.818 μm, 1.021 μm, and 0.961 μm, respectively.

The adhesion and biocompatibility of BMSCs on various scaffolds were evaluated.

Cells were cultured on P, PH, and PH/PDA scaffolds and stained with phalloidin as well as Calcein AM and propidium iodide at designated time points. Confocal laser scanning microscopy (CLSM) was then employed to observe cell adhesion and viability.

Morphological images of BMSCs were obtained after 2 and 24 h of culture. At 2 h, the cells had adhered to the scaffold surfaces. After 24 h, rBMSCs on the PH/PDA scaffold exhibited a more extensively spread morphology compared with those on the P and PH scaffolds (Fig. S5A, Supporting Information). Quantitative analysis of cell area further confirmed that the incorporation of nHap and PDA facilitated cell adhesion (Fig. S5B, Supporting Information). Moreover, compared to the P and PH scaffolds, the BMSCs on the PH/PDA scaffold demonstrated enhanced faster growth rate and uniform distribution (Fig. 4H–and Figure S5C and D, Supporting Information). These findings were further confirmed by CCK-8 cell viability assays (Fig. 4I).

We evaluated the release profile of h-EXOs from the scaffold and found that they could be continuously released over time up to day 20. Meanwhile, transmission electron microscopy (TEM) analysis was performed on h-EXOs collected between days 17 and 20, revealing that the exosomes still maintained their structural integrity (Fig. S6, Supporting Information). In addition, co-culture of HUVECs and BMSCs with h-EXOs released from the PH/PDA/h-EXOs scaffold at a late stage (day 20) demonstrated that the released h-EXOs retained good biological activity (Fig. S7, Supporting Information).

### 2.5 PH/PDA/h-EXOs scaffold promotes the angiogenic capacity of HUVECs and M2 polarization of macrophages

**Fig. 5:**
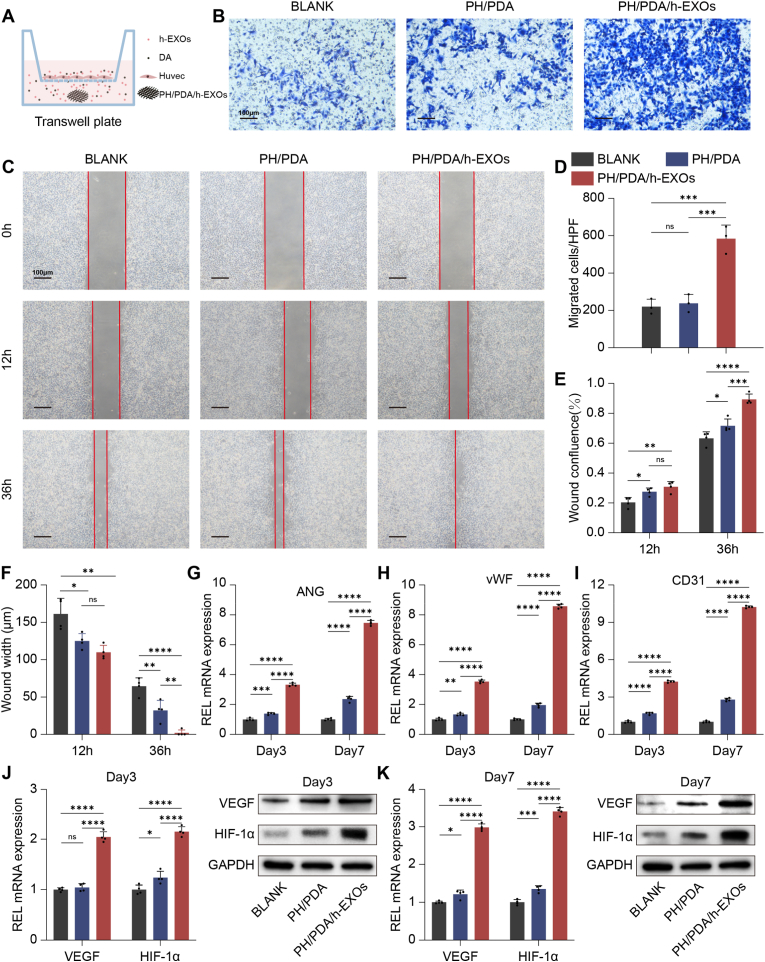
**In vitro angiogenic potential of PH/PDA/EXO scaffold. A.** Schematic illustration of transwell co-culture system. **B.** Representative images of the transwell migration assay at 24 h. **C.** Wound healing microscopic images of HUVECs treated with different scaffolds at 0, 12, and 36h. **D.** Quantitative analysis of migrated HUVECs. (n = 3) **E-F.** Wound closure percentage and wound width determined by microscopic images. (n = 4) **G-I.** Expression levels of angiogenesis-related genes evaluated by qRT-PCR on days 3 and 7 of co-culture. (n = 4) **J-K.** Expression levels of VEGF and HIF-1α genes assessed by qRT-PCR and WB on days 3 and 7 of co-culture. (n = 4) ∗p < 0.05, ∗∗p < 0.01, ∗∗∗p < 0.001, ∗∗∗∗p < 0.0001.

The angiogenic potential of scaffolds is a key determinant of successful bone repair. First, Calcein-AM/PI staining and CCK-8 assays demonstrated that the PH/PDA scaffold exhibits good biocompatibility, and the incorporation of h-EXOs further promotes the proliferation of HUVECs (Fig. S8, Supporting Information). A Transwell assay was conducted to evaluate the potential chemotactic effects of the scaffold on HUVECs (Fig. 5A). As displayed in Fig. 5B, the release of h-EXOs from the scaffolds significantly promoted the recruitment of HUVECs after 24 h of co-culture, particularly in the PH/PDA/h-EXOs scaffold group (Fig. 5D). Besides, a scratch assay was performed to assess the migratory ability of cells in response to h-EXOs stimulation. As shown in Fig. 5C, cell migration was markedly increased in both the PH/PDA and PH/PDA/h-EXOs groups compared to the control group. The PH/PDA/h-EXOs group achieved the most effective wound closure at 36 h, with the scratch nearly closed, as confirmed by quantitative analysis (Fig. 5E and F). Tube formation assays demonstrated that the presence of h-EXOs promoted the rapid formation of capillary-like structures within 6 h (Fig. S9A, Supporting Information). Compared with the blank control group and the scaffold-only group, the h-EXOs-loaded scaffold exhibited significantly increased tube length and branch node numbers, indicating markedly enhanced angiogenic capacity (Fig. S9B and C, Supporting Information). Overall, these findings suggest that the PH/PDA/h-EXOs composite scaffold significantly enhances cell migration and angiogenesis.

To assess the effects of different scaffolds on angiogenesis at the molecular level, the expression of key angiogenic genes, such as angiopoietin (ANG), von Willebrand factor (vWF), and CD31 were significantly increased in cells cultured with h-EXOs-loaded scaffolds (Fig. 5G–I). Furthermore, HIF-1α and VEGF, was analyzed by qRT-PCR and WB after 3 and 7 days of induction. At both time points, the PH/PDA/h-EXOs scaffold exhibited the highest expression levels of angiogenesis-related genes (Fig. 5J and K).

h-EXOs heve been proven to have the potential to stimulate M2 polarization of macrophages; then, PH/PDA/h-EXOs scaffolds also need to be verified. qRT-PCR was used to assess the expression of macrophage M2/M1 phenotypic markers. The results clearly show that the PH/PDA/h-EXOs scaffold significant upregulation of M2-associated markers, accompanied by decreased expression of M1-related markers (Fig. S10, Supporting Information).

### 2.6 PH/PDA/h-EXOs scaffold promotes the osteogenic capacity of BMSCs

**Fig. 6:**
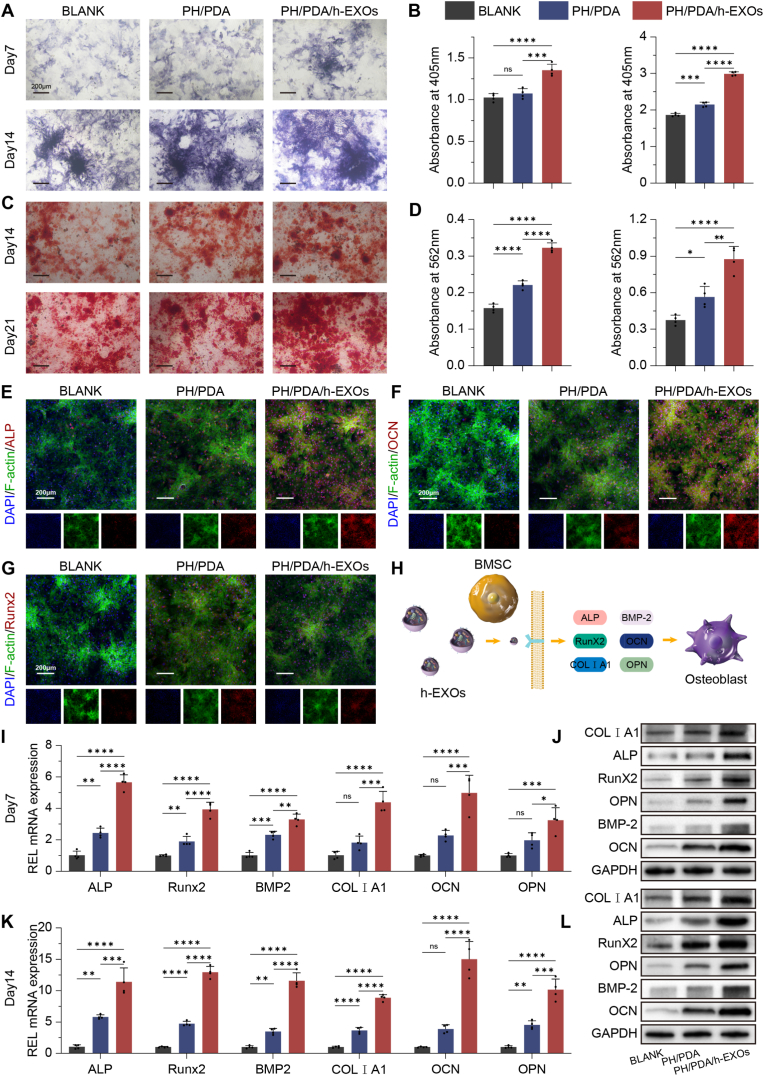
**In vitro osteogenic potential of PH/PDA/EXO scaffold. A-B.** ALP staining images and quantitative analysis after osteogenic induction with different scaffolds on days 7 and 14. (n = 4) **C-D.** ARS staining images and quantitative analysis after osteogenic induction with different scaffolds on days 14 and 21. (n = 4) **E-G.** Immunofluorescence (IF) staining of osteogenic markers ALP, OCN and Runx2 in BMSCs induced by different scaffolds. **H.** The mechanism of osteogenic gene activation by PH/PDA/h-EXOs. **I-L.** Osteogenic markers ALP, Runx2, BMP-2, COLⅠA1, OCN, and OPN assessed by qRT-PCR and WB at days 7 and 14. (n = 4) ∗p < 0.05, ∗∗p < 0.01, ∗∗∗p < 0.001, ∗∗∗∗p < 0.0001.

Given that osteoblast differentiation is a key factor influencing the efficiency of bone regeneration, the osteogenic activity of various scaffolds was systematically evaluated using ALP staining, Alizarin Red S (ARS) staining, qRT-PCR, and WB. As shown in Fig. 6A, on days 7 and 14, the surfaces of the PH/PDA and PH/PDA/h-EXOs scaffolds exhibited more prominent blue staining compared to the control group, indicating that the combination of nHap, PDA, and h-EXOs synergistically enhanced osteogenic performance. Likewise, quantitative analysis of ALP activity further revealed that the PH/PDA/h-EXOs group exhibited the highest ALP activity, reflecting its superior osteogenic potential (Fig. 6B). Consistent results were obtained from ARS staining, as presented in Fig. 6C and D. Afterward, the osteogenic capacity of the PH/PDA/h-EXOs scaffold was assessed using immunofluorescence analysis (IF), yielding results consistent with the staining assays. Specifically, the expression of the osteoblast marker gene ALP, the master regulator of osteoblast differentiation transcription factor runt-related transcription factor 2 (Runx2), and the late-stage osteogenic marker OCN highlighted the ability of the PH/PDA/h-EXOs scaffold to induce BMSCs differentiation (Fig. 6E–F).

Additionally, qRT-PCR assays were carried out to assess the expression of osteogenic genes across different scaffold groups, including ALP, Runx2, bone morphogenetic protein-2 (BMP-2), collagen, type I, alpha 1 (Col IA1), OCN, and osteopontin (OPN) [42]. The expression levels of these osteogenic markers increased in the PH/PDA and PH/PDA/h-EXOs groups on the 7th and 14th days (Fig. 6I and K). Moreover, the expression levels of the late-stage markers OCN and OPN significantly increased in the presence of h-EXOs at day 14. WB analysis of BMSCs cultured for one and two weeks unveiled that the PH/PDA/h-EXOs group exhibited markedly darker and broader bands compared to the other groups, indicating a significant difference (Fig. 6J and L). Overall, these results indicate that the incorporation of h-EXOs in the PH/PDA scaffold significantly enhanced the scaffold's capacity to promote the osteogenic differentiation of BMSCs. A schematic illustration of the activation of osteogenic genes by PH/PDA/h-EXOs is illustrated in Fig. 6H.

### 2.7 PH/PDA/h-EXOs scaffold promotes osteogenic differentiation through the NGF/PI3K/AKT pathway

**Fig. 7:**
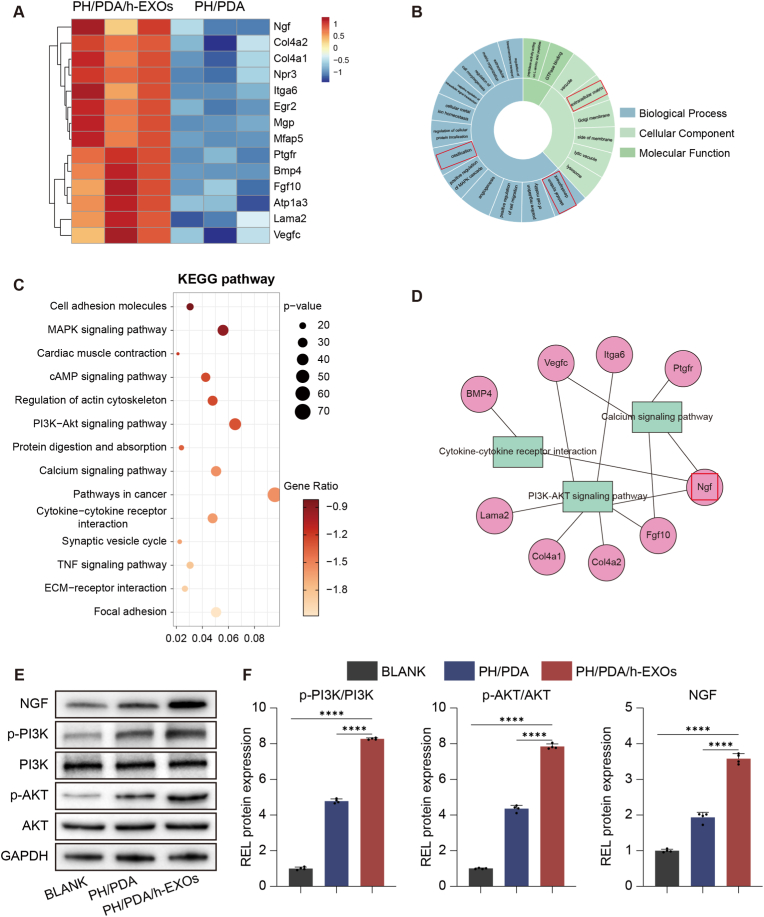
**Mechanistic analysis of h-EXOs promote osteogenesis. A.** Heatmap visualization of osteogenesis-related differential gene expression. **B-C.** 20 enriched GO terms and 14 KEGG pathways in BMSCs cultured with PH/PDA and PH/PDA/h-EXOs induction media. **D.** Genes maps related to cytokine-cytokine–receptor interaction, PI3K/AKT signaling pathway, and calcium signaling pathway. **E-F.** Expression and quantification of NGF and key proteins in the PI3K/AKT signaling pathway in BMSCs cultured on different scaffolds for 7 days, analyzed by WB. (n = 4) ∗∗∗∗p < 0.0001.

To elucidate the potential mechanisms by which the composite scaffolds enhance osteogenic activity, whole-transcriptome RNA sequencing was conducted on BMSCs cultured with PH/PDA and PH/PDA/h-EXOs scaffolds. In this study, based on DIA criteria (P ≤ 0.05 and log2FC ≥ 0.585), a total of 1362 upregulated genes and 473 downregulated genes were identified, which were visualized through a volcano plot (Fig. S11, Supporting Information). Fourteen upregulated genes associated with osteogenesis are listed in Fig. 7A. The differentially expressed genes were then subjected to GO analysis, with the 20 significantly enriched terms shown in Fig. 7B. The results indicated that, compared to the PH/PDA scaffold, the addition of h-EXOs regulated ossification, skeletal system development, angiogenesis, and the extracellular matrix. Among these, extracellular matrix (ECM)-related genes exhibited patterns similar to those of ECM-receptor interaction pathways identified in the KEGG pathway enrichment analysis (Fig. 7C). In addition, pathways associated with osteogenesis, such as the PI3K/AKT signaling pathway and the focal adhesion pathway, were significantly enriched. Importantly, NGF, an osteogenic gene upregulated as shown in Fig. 7D, was linked to all three of these pathways. Considering that previous studies have established the role of the PI3K/AKT pathway in regulating angiogenesis and bone metabolism, we speculate that NGF may enhance osteogenic activity in the PH/PDA/h-EXOs scaffold system through activation of this pathway [43,44]. To further investigate the effect of the composite scaffold on the NGF/PI3K/AKT signaling pathway, WB analysis was performed (Fig. 7E). As illustrated in Fig. 7F, the expression levels of phosphorylated PI3K (p-PI3K) and AKT (p-AKT) were significantly higher in both the PH/PDA and PH/PDA/h-EXOs groups compared to the blank group, with the most substantial increase observed in the PH/PDA/h-EXOs group.

We used capivasertib, a potent PI3K/AKT pathway inhibitor, to investigate the role of the PI3K/AKT pathway in h-EXO–mediated osteogenic differentiation in vitro. Specifically, ALP staining showed that, after 7 days, the staining area was markedly reduced in the presence of capivasertib, indicating that the pro-osteogenic effect of h-EXOs was significantly attenuated (Fig. S12A, Supporting Information). Runx2 immunofluorescence staining showed a similar trend (Fig. S12B, Supporting Information). In addition, qRT-PCR demonstrated that h-EXOs significantly upregulated the expression of osteogenic genes, including ALP and Runx2, whereas capivasertib largely reversed these effects (Fig. S12C and D, Supporting Information). Consistently, Western blot analysis further confirmed that capivasertib suppressed PI3K/AKT pathway activation and reduced the expression of osteogenesis-related proteins (Fig. S12E, Supporting Information). Meanwhile, h-EXOs significantly enhanced the expression of angiogenesis-related genes in HUVECs, and this effect was reversed by the PI3K/AKT pathway inhibitor capivasertib (Fig. S13, Supporting Information).

In addition, based on our RNA-sequencing results, we further identified the cAMP signaling pathway as another significantly enriched pathway associated with the biological effects of h-EXOs. Therefore, we employed the adenylate cyclase inhibitor SQ22536 and found that blocking the cAMP signaling pathway attenuated the osteogenic and pro-angiogenic effects of h-EXOs (Fig. S14, Supporting Information).

### 2.8 PH/PDA/h-EXOs scaffold enhances bone regeneration in vivo

**Fig. 8:**
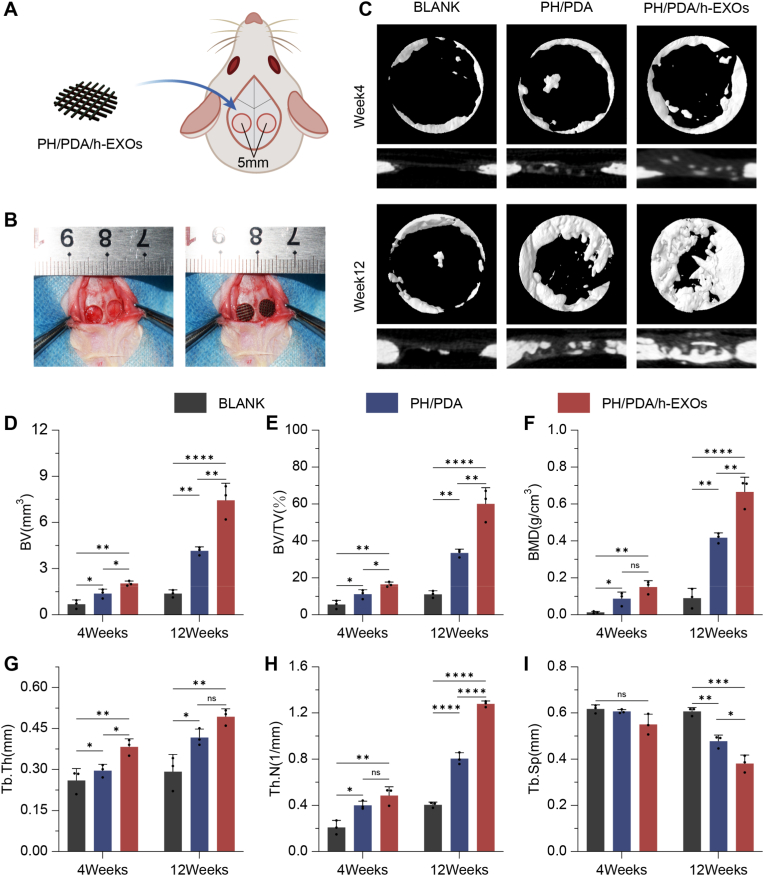
**PH/PDA/h-EXOs scaffold could enhance bone regeneration in vivo. A.** Schematic illustration of cranial defect repair. **B.** Intraoperative images of the implantation site. **C.** Micro-CT 3D reconstruction images of bone defects from different angles at 4 and 12 weeks following scaffold implantation. **D-I.** Quantitative analysis of BV, BV/TV, BMD, Tb.Th, Tb.N and Tb.Sp from 3D micro-CT images at weeks 4 and 12. (n = 3) ∗p < 0.05, ∗∗p < 0.01, ∗∗∗p < 0.001, ∗∗∗∗p < 0.0001.

To assess the osteogenic capacity of the composite scaffold in vivo, a critical-sized (5 mm) skull defect model was established in rats (Fig. 8A and B). Micro-CT imaging was utilized to quantify new bone formation within the defect regions. Evidence of early bone reformation was noted in the scaffold-implanted groups at 4 weeks. By 12 weeks, CT scans revealed distinct differences in regenerative outcomes across groups (Fig. 8C). Specifically, the PH/PDA/h-EXOs group exhibited substantial defect closure with well-integrated, continuous new bone formation. In contrast, the PH/PDA group displayed only partial defect bridging, with significant voids observed. As expected, the untreated BLANK group demonstrated minimal bone regeneration.

Quantitative Micro-CT analysis validated these observations: both BV and BV/TV were significantly higher in the PH/PDA/h-EXOs group compared to the blank and blank PH/PDA groups, especially at week 12 (Fig. 8D–F). Meanwhile, key trabecular bone indices in the PH/PDA/h-EXOs group, including trabecular thickness (Tb.Th), separation (Tb.Sp), and number (Tb.N), also showed marked improvement (Fig. 8G–I). Notably, this group exhibited the highest BMD, highlighting the scaffold's capacity to support robust osteogenesis. These findings conjointly indicate that h-EXOs-loaded scaffolds effectively promote bone regeneration in vivo.

### 2.9 Histological, immunohistochemical, and immunofluorescent analysis of new bone tissue in vivo

**Fig. 9:**
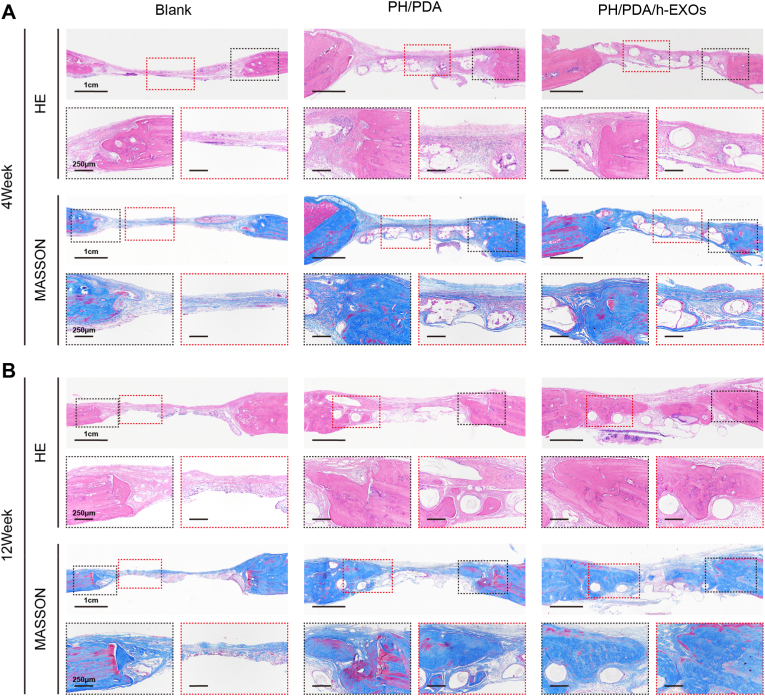
**Histological analysis of new bone tissue in vivo. A.** H&E and masson's trichrome staining at week 4. **B.** H&E and masson's trichrome staining at week 12.

At weeks 4 and 12, histological evaluations using hematoxylin and eosin (H&E) staining and Masson's trichrome staining were consistent with the results of micro-CT imaging. Following implantation, newly formed bone was observed at the defect margins of the PH/PDA/h-EXOs scaffold, indicating a high level of osteoconductivity at week 4 (Fig. 9A). By 12 weeks post-implantation, the h-EXOs-loaded PH/PDA scaffolds significantly promoted new bone formation within the core region, as evidenced by the extensive growth of new bone along the scaffold structure (Fig. 9B). In contrast, the blank and PH/PDA groups showed substantial tissue loss and minimal matrix deposition in the center of the defect.

**Fig. 10:**
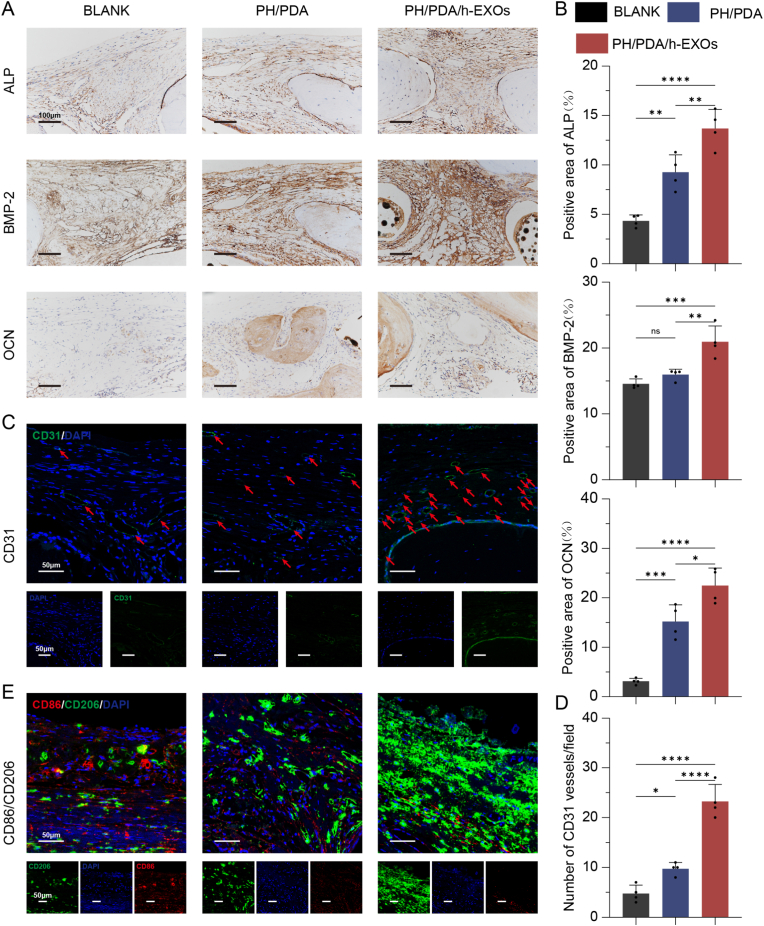
**Immunohistochemical, and immunofluorescent analysis of new bone tissue in vivo. A-B.** Representative images and quantitative analysis of immunohistochemical staining for ALP, BMP-2, and OCN. (n = 4) **C-D.** Immunofluorescence staining and quantitative analysis of CD31 (Green) and nucleus (Blue) in defect tissue sections. (n = 4) **E.** Representative images of CD86 (Red) and CD206 (Green) in tissue sections, with nucleus counterstained with DAPI (Blue) in all images. ∗p < 0.05, ∗∗p < 0.01, ∗∗∗p < 0.001, ∗∗∗∗p < 0.0001. (For interpretation of the references to color in this figure legend, the reader is referred to the Web version of this article.)

To further assess osteogenic activity, immunohistochemical staining for ALP, BMP-2, OCN, and Runx2 was performed (Fig. 10A, and Fig. S15, Supporting Information). Quantitative analysis revealed significantly higher expression levels of osteogenic markers in the PH/PDA/h-EXOs group, indicating enhanced osteoblastic differentiation and bone formation (Fig. 10B). Moreover, the PH/PDA/h-EXOs group exhibited markedly stronger p-AKT and p-CREB expression than the control groups (Fig. S16A and B, Supporting Information). Quantitative analysis further confirmed that h-EXOs significantly promoted AKT and CREB phosphorylation in vivo, indicating activation of the PI3K/AKT and cAMP signaling pathway at the bone defect site (Fig. S16C and D, Supporting Information). Concurrently, immunofluorescent staining for CD31, a marker of endothelial cells, demonstrated that the introduction of h-EXOs promoted angiogenesis compared to the other 3D-printed groups (Fig. 10C and D). In addition, immunofluorescence staining uncovered the presence of both CD86 (red fluorescence, M1 macrophages) and CD206 (green fluorescence, M2 macrophages) across all three groups (Fig. 10E). Lastly, quantitative analysis showed that the CD206/CD86 ratio was significantly higher in the PH/PDA/h-EXOs scaffold group compared to the other groups, indicating that the presence of h-EXOs promoted a shift toward the M2 macrophage polarization (Fig. S17, Supporting Information).

## 3 Discussion

The repair of large bone defects remains a significant challenge in the clinical setting. The cross-talk between organs within the body has provided valuable insights into bone repair, with exosomes offering significant bioactivity and scaffolds providing a promising loading platform. Herein, exosomes were isolated from hepatocytes using a commercial kit. Subsequently, h-EXOs were loaded onto the surface of the DLP-printed PH scaffolds coated with PDA. These scaffolds exhibited satisfactory biological responses in vitro. Finally, the scaffolds loaded with h-EXOs demonstrated promising osteogenic capacity in cranial bone defects.

As two critical metabolic organs, the cross-talk between the bone and liver holds critical implications for both scientific research and clinical applications [19]. Several clinical studies have demonstrated that hepatic function can influence bone quality. For example, liver diseases have been reported to increase the risk of osteoporosis and fracture [45,46]. Meanwhile, a large number of studies have explored the cross-talk between the liver and bone. Wang et al. discovered that liver-secreted leucine-rich α-2-glycoprotein 1 regulates liver-bone communication and promotes bone mineralization [47]. Lee et al. pointed out that undercarboxylated osteocalcin secreted by bone tissue enhances hepatic insulin sensitivity and modulates energy expenditure [48]. These findings corroborate the presence of a cross-talk between the liver and bone, promoting the dynamic balance between the two organs. Exosomes, as important mediators of intercellular communication, have recently garnered widespread attention owing to their excellent targeting efficiency, rich biological activities, and low immunogenicity. Chen et al. utilized miR-183-5p, abundantly expressed in hepatocytes, to negatively regulate the expression of the target gene FoxO1, thereby promoting liver regeneration and restoring hepatic function [49]. According to an earlier study, h-EXOs exert effects beyond the liver. For instance, selenoprotein P secreted by the liver can cross the blood-brain barrier and reach neurons via h-EXOs-mediated transport [50]. Through inter-organ interactions, h-EXOs can exert distinct effects in various tissues.

RNA sequencing revealed that h-EXOs are enriched in signaling molecules capable of modulating cellular pathways, thereby altering cellular states. Consequently, h-EXOs were co-cultured with BMSCs and endothelial cells, resulting in elevated expression levels of genes associated with angiogenesis and osteogenic differentiation. Additionally, previous studies have pointed out that h-EXOs inhibit the secretion of inflammation-related cytokines [38]. Herein, hepatocyte-derived exosomes promoted the differentiation of M0-type macrophages into M2-type macrophages, thereby alleviating inflammation at the site of injury.

Polycaprolactone (PCL) has been widely used in clinical and tissue engineering applications. Its excellent mechanical properties and prolonged degradation period enable the scaffold to remain stably fixed at the defect site for an extended duration, thereby preventing structural collapse. Moreover, its favorable biocompatibility minimizes disruption to the local microenvironment at the injury site [51,52]. However, the commonly used printing method for PCL, namely fused deposition modeling (FDM) has been linked to relatively low printing accuracy [53]. In contrast, DLP printing is highly suitable for fabricating tissue-engineering scaffolds, attributable to its ultra-high accuracy, high efficiency, and customizability [35,54]. Additionally, inspired by the photoreactive nature of hydrogels, PCLMA was selected for high-precision printing [37]. However, PCL is highly hydrophobic, which limits cell adhesion and bioactivity, thereby limiting bone tissue regeneration and potentially increasing the risk of implant loosening or premature failure [39,55]. In this study, the PH/PDA/h-EXOs scaffold exhibited favorable biocompatibility, which may be ascribed to the following modifications: 1. The incorporation of nHap provides additional binding sites for cell adhesion, enhancing the surface roughness of the scaffold, promoting cellular attachment, and supporting cell proliferation. More importantly, the incorporation of nHap did not significantly compromise the printing accuracy of PCLMA. 2. The application of a uniformly distributed PDA coating on the scaffold significantly improved its hydrophilicity, enhanced vesicle loading, promoted cell adhesion on the scaffold, and ultimately improved the biological performance of the scaffold [56,57]. 3. The even distribution of nHap and PDA promoted the uniform release of calcium and phosphate ions, as well as dopamine, which in turn facilitated stem cell migration toward the bone defect area and enhanced osteogenesis.

Angiogenesis plays an instrumental role in bone repair [[58], [59], [60]]. Blood vessels secrete various factors, such as VEGF and PDGF-BB, thereby driving angiogenesis and the recruitment of surrounding osteoblasts to facilitate bone repair [61]. In the present study, the PH/PDA/h-EXOs scaffold enhanced the migratory capability of HUVECs and up-regulated the expression of multiple angiogenic and osteogenic genes in vitro. Meanwhile, the local inflammatory response plays a crucial role in bone tissue regeneration, and an appropriate level of inflammation can promote bone healing [62]. Recent studies have shown that the proportion of M2-polarized macrophages increases during fracture repair, and these cells induce osteogenic differentiation by secreting various growth factors [63]. Therefore, accelerating the transition from M1 to M2 macrophages can enhance early recruitment of osteogenic cells and promote angiogenesis. In this study, the PH/PDA/h-EXOs scaffold was able to rapidly promote the polarization of M1 macrophages toward the M2 phenotype in vitro, thereby modulating the osteogenic microenvironment. Furthermore, the presence of h-EXOs markedly facilitated BMSC osteogenesis, with significantly higher expression levels of ALP, OCN, and OPN compared to scaffolds lacking exosomal stimulation. It is worthwhile emphasizing that RNA sequencing indicated that after the addition of h-EXOs (excluding oncogenesis-related pathways), the top three pathways enriched with up-regulated genes were related to NGF. Gu et al. identified a close correlation between high activity of ECM-receptor interaction pathways and fracture healing [64]. Vermeulen et al. documented that expanding the design space of TopoChip along a natural plane impacted the alignment of actin filaments and focal adhesions, thereby modulating the differentiation potential of mesenchymal stem cells [65]. The PI3K/AKT pathway plays a major role in mediating angiogenesis and bone tissue metabolism. Shi et al. designed a composite hydrogel containing calcium phosphate oligomer and BMP-2 that can activate the ITGA10/PI3K/AKT signaling pathway, thereby promoting osteogenic gene expression in BMSCs and facilitating bone repair [66]. Integrating these results with the findings of Western blot analysis suggests that h-EXOs may promote osteogenesis in BMSCs by activating the NGF/PI3K/AKT signaling pathway, while simultaneously influencing ECM-receptor interactions and focal adhesion.

To validate the osteogenic efficacy of h-EXOs, a cranial bone defect model was generated. Interestingly, the defect area in the PH/PDA/h-EXOs group was almost completely filled by newly formed, dense bone tissue 12 weeks after implantation. The potential in vivo bone regeneration process can be summarized as follows: The defect area is populated with mesenchymal stem cells, progenitor cells, osteoblasts, and endothelial cells [67]. Upon implantation at the defect site, the PH/PDA/h-EXOs scaffold reduces the proportion of M1 macrophages and enhances polarization towards M2 macrophages around the scaffold, while simultaneously promoting angiogenesis. These modifications collectively ameliorate the microenvironment at the defect site, thereby promoting early stem cell differentiation and accelerating new bone tissue formation between the defect margins and the scaffold. Over time, with sustained nutrient and blood supply, newly formed bone tissue progressively deposits on the scaffold, eventually interconnecting and nearly filling the entire defect area. Of note, h-EXOs play a crucial role throughout this process, including promoting angiogenesis, modulating immune responses, and facilitating osteogenesis.

In summary, this study leveraged inter-organ cross-talk to develop a novel approach for bone repair. By activating the NGF/PI3K/AKT signaling pathway, h-EXOs effectively induce osteogenic differentiation in vitro and hold substantial promise for promoting bone regeneration in vivo. The findings provide direct evidence supporting the presence of cross-talk between the liver and bone. However, several aspects warrant further exploration. Future studies are necessitated to further investigate the mechanisms by which different types of bone defects influence hepatic signaling pathways and the corresponding secretory profiles. In addition, the scaffold design can be further optimized according to specific defect conditions, including its architecture, porosity, and mechanical properties. Meanwhile, the influence of PDA coating thickness and the loading and release strategies of h-EXOs in different applications should be further investigated to enhance regenerative efficacy. Furthermore, the relationship between the long-term degradation rate of the scaffold and bone remodeling requires more systematic evaluation. Finally, the present study design lays a theoretical reference for the exploration of vesicles derived from other organs as therapeutic agents for tissue repair.

## 4 Conclusion

In this study, based on the interactions between organs, we extracted hepatocyte-derived exosomes using a commercial kit and demonstrated their ability to promote osteogenesis, angiogenesis, and immune regulation. We further loaded these vesicles onto a customized, PDA-treated PH scaffold, which exhibited superior bone ingrowth capabilities compared to other scaffolds. Through RNA sequencing and WB, we identified and validated that exosomes secreted by hepatocytes can enhance BMSCs differentiation through activation of the NGF/PI3K/AKT signaling pathway, thereby influencing bone formation. Moreover, the autologous transplantation of vesicles significantly reduced immune rejection, presenting promising prospects for future clinical applications.

## 5 Method

### 5.1 Cells isolation and culture

Primary hepatocytes were isolated from SD rats (150-200 g) via a two-step collagenase perfusion method [47]. Briefly, the liver was perfused through the portal vein with pre-warmed (37°C) perfusion buffer (HBSS without Ca2+/Mg2+, Thermo Fisher, 14175092; containing 0.5 mM EDTA, Thermo Fisher, AM9260G) for 5 min, followed by digestion buffer (HBSS with Ca2+/Mg2+, Thermo Fisher, 14025095; containing 0.5 mg/mL type IV collagenase, Thermo Fisher, 17104019) for 10-15 min. The softened liver was dissociated by gentle agitation, filtered through a 70 μm cell strainer, and centrifuged at 100 ×g for 5 min. Hepatocytes were resuspended in Williams’ E medium supplemented with 10% FBS and seeded at a density of 5×10^4 cells/cm2.

BMSCs were isolated from 5-day-old male SD rats following a previously established protocol [68]. Specifically, the neonatal rats were euthanized via spinal dislocation and subsequently submerged in 75% ethanol for a 10-min disinfection period. The femora and tibiae were then carefully excised. The cartilaginous ends of the bones were removed. Bone marrow cavities were thoroughly flushed with 10 mL of culture medium. The collected bone marrow was then seeded into a culture dish containing 8 mL of culture medium (L-DMEM supplemented with 10% fetal bovine serum (FBS) and 1% penicillin/streptomycin). The culture dishes were incubated at 37°C, with medium changes performed every 3 days. BMSCs at passages 3-5 (confirmed by CD90+/CD29+ expression >90% via flow cytometry) were used for experiments.

### 5.2 H-EXOs isolation and determination

h-EXOs were isolated from hepatocyte culture supernatant using the exoEasy Maxi Kit (QIAGEN, Germany). Briefly, the supernatant was sequentially centrifuged at 300 ×g for 10 min, 2000 ×g for 20 min, and filtered through a 0.45 μm membrane. The clarified supernatant (1 mL) was processed according to the kit protocol, and h-EXOs were eluted in 100 μL Elusion buffer. The morphology and size of h-EXOs were assessed by TEM and ZetaView (Particle Metrix, Germany). The presence of EXO markers was confirmed via WB analysis to verify the successful isolation of EXO. We selected 25 μg/mL as the primary concentration for most co-culture experiments involving HUVECs and BMSCs.

### 5.3 Western blot assay

Proteins were isolated from HUVECs and BMSCs via the RIPA lysis method, and quantified by BCA assay. Equivalent quantities of protein were loaded onto SDS-PAGE and transferred to PVDF membranes. After blocking with 5% (w/v) skimmed milk solution at room temperature for 1 h. Subsequently, the samples were incubated with primary antibodies (Abcam) at a dilution of 1:1000 for CD63 (ab134045), CD81 (ab109201), TSG101 (ab133586), Calnexin (ab227310), HIF-1α (ab179483), VEGF (ab32152), ALP (ab229126), Runx2 (ab92336), BMP-2 (ab284387), OCN (ab133612), and OPN (ab283656) overnight at 4°C. Membranes were then incubated with HRP-conjugated secondary antibodies (anti-rabbit and anti-mouse). The immune complexes were visualized using the Pro-Light horseradish peroxidase (HRP) Kit.

### 5.4 qRT-PCR assay

RNA was isolated from macrophages, BMSCs, and HUVECs using the TRIzol reagent. Quantitative qRT-PCR was employed to determine the relative expression levels of RNA, with glyceraldehyde-3-phosphate dehydrogenase (GAPDH) serving as the reference gene for normalization. The PCR reactions were carried out in a final volume of 20.0 μL. The primer sequences are provided in Table S1 qPCR was performed in 20 μL reactions containing Hieff SYBR Green Master Mix (Yeason, 11201ES03) over 40 cycles. Data were analyzed by the 2^−ΔΔCt^ method.

### 5.5 H-EXOs labeling and tracking

Purified h-EXOs were labeled with PKH26 (Umibio, UR52302), following the manufacturer's protocol. BMSCs were cultured with PKH26-h-EXOs in serum-free DMEM for 12 h at 37°C. Cells were then fixed with 4% PFA, permeabilized with 0.1% Triton X-100, and stained with 488-phalloidin (1:200, Invitrogen) and Hoechst 33342 (5 μg/mL, Beyotime). The uptake of the labeled exosomes by the cells was imaged using a confocal microscope (Nikon, Tokyo, Japan).

### 5.6 Fabrication of PCLMA-nHap (PH) scaffold

A three-dimensional bone scaffold model was designed in UG12.0 software and produced via digital light processing (DLP) 3D printing (EFL-BP8601 Pro printer). The construct dimensions (5 mm diameter × 0.5 mm height) were optimized for cranial critical-sized defects. For material preparation, nano-hydroxyapatite (nHap, 10 wt%) was uniformly dispersed in polycaprolactone methacrylate (PCLMA) resin prior to printing. Post-fabrication, the PH scaffolds underwent three sequential ultrasonic cleaning cycles (25-30 s each) using a 1:1 methanol/isopropanol solution (Fisherbrand ultrasonic cleaner, 20-35 kHz)

### 5.7 Enzyme-linked immunosorbent assay

RAW264.7 cells cultured in 24-well plates were treated with PBS, LPS (100 ng/mL), LPS (100 ng/mL) + h-EXOs (100 μg/mL) for 24 h After 24 h of incubation, supernatant samples from each group were collected. Concentrations of IL-1β, TNF-α, and IL-10 were quantified using commercial ELISA kits (Sigma-Aldrich) following the manufacturer's protocols. Air pouch fluid samples were similarly processed for cytokine detection.

### 5.8 The hydrophilicity of PCLMA

The WCA of the P, PH, and PH/PDA wafers was measured to evaluate their surface hydrophilicity using the sessile drop method. Using the sessile drop technique, 2.0 μL deionized water droplets were precisely dispensed onto each material's surface with a microsyringe. Contact angle values were determined by analyzing high-resolution images captured during testing, employing a commercial drop shape analyzer (DSA100, Krüss, Germany).

### 5.9 The morphological analysis of scaffolds

The AFM (SPM9700, Shimadzu) was utilized to assess the microscopic characteristics of the P, PH, and PH/PDA wafers. The Gemini 300 (Zeiss) was employed to capture images. A 30-s gold-sputtering process was performed on P, P/PDA, PH, PH/PDA, and PH/PDA/h-EXOs scaffold samples, followed by scanning at 5 kV. Additionally, a laser scanning confocal microscope (LSCM) (OLS5000, Olympus) was used to evaluate the microscopic features of the P, PH, and PH/PDA scaffolds.

### 5.10 Biocompatibility test of the scaffold

The cytotoxicity and cell growth on P, PH, and PH/PDA scaffolds were evaluated through CCK-8 proliferation assays and live/dead staining. The scaffolds were sterilized by ultraviolet irradiation and ozone treatment. Subsequently, cells (2 × 10^4 per well) were seeded onto the scaffold surfaces (n = 4). After an initial 2-h attachment period, each scaffold was transferred to a new 24-well plate and incubated with 1 mL of freshly prepared medium per well. At various time points (1, 3, and 7 days), cellular metabolic activity was determined by incubating with CCK-8 reagent (10% v/v, Dojindo) for 2 h, followed by absorbance measurement at 450 nm (Thermo Microplate Reader).). Viability was simultaneously assessed using fluorescent markers: calcein AM (2 μM) for viable cells and ethidium homodimer-1 (4 μM) for non-viable populations. Cell distribution and morphology were visualized by laser scanning confocal microscopy (Nikon A1R) at matching time points.

### 5.11 Cell migration assay

Cell migration was assessed using both scratch healing assays and transwell assays. Cells (30,000/insert) in serum-free medium (200 μL) were seeded into the upper chambers (8 μm pores, Corning) with complete medium (10% FBS) in the lower compartment. After 24-h incubation (37°C, 5% CO_2_), migrated cells were fixed with 4% paraformaldehyde (30 min, RT), and non-migrated cells on the upper membrane surface were removed mechanically. Cells were stained with 0.01% crystal violet (15 min) and imaged under a phase-contrast microscope (Olympus BX51, 200×). Migrated cells in five random fields per insert were counted using ImageJ (n = 3 independent experiments).

For the scratch wound healing assay, HUVECs (90% confluence, 1.5×10^6 cells/well in 6-well plates) were scratched with a sterile 200 μL pipette tip. After PBS washing, cells were maintained in complete medium. Wound closure was monitored at 0, 12, and 36 h using an inverted microscope (Nikon Eclipse Ts2R-FL). The relative wound width was quantified using ImageJ's "MRI Wound Healing Tool" plugin (n = 3, with triplicate wells per experiment).

### 5.12 ALP activity and ARS staining

The osteoinductive capacity of P, PH, and PH/PDA matrices was investigated by culturing BMSCs in osteogenic differentiation medium. Alkaline hosphatase (ALP) expression was analyzed through both qualitative staining and quantitative activity measurements.For histological assessment, cellular ALP was detected using a BCIP/NBT colorimetric kit (Beyotime, C3206). Quantitative analysis was performed with a commercial ALP activity assay (Beyotime, P0321S) following the manufacturer's protocol. The procedures were carried out according to the manufacturer's instructions, and absorbance was recorded at 405 nm using a microplate reader (ELX-800, BioTek, USA). ARS staining was employed to visualize calcium nodule formation in BMSCs. Calcium nodules were assessed using ARS (Solarbio, China). For quantification, the stained cells were washed three times with deionized water, eluted with 10% cetylpyridinium chloride for 10 min, and the absorbance was measured at 562 nm.

### 5.13 RNA-Seq analysis

Transcriptome profiling was performed by APPLIED PROTEIN TECHNOLOGY (Shanghai, China) using Illumina sequencing platforms. Total RNA was extracted from BMSCs cultured on PH/PDA and PH/PDA/h-EXOs substrates using RNA-easy isolation reagent (Vazyme, R701-01). RNA integrity was verified by Agilent 2100 bioanalyzer (RIN >7.0). Library preparation was conducted following the manufacturer's protocol, and paired-end sequencing (150 bp) was performed on Illumina NovaSeq 6000 platform.

### 5.14 Repair of bone defects

This study utilized sixteen 6-week-old SD rats, with all procedures approved by the Ethics Committee of Huazhong University of Science and Technology and Institutional Animal Care and Use Committee (IACUC) number [4726]. Animals were randomly assigned to three groups using a random number table: (1) Skull defect only (Blank group, n = 5), (2) PH scaffolds modified with PDA (PH/PDA group, n = 5), (3) PH/PDA scaffolds incorporating h-EXOs (PH/PDA/h-EXOs group, n = 5). All evaluations-including micro-CT analysis, histological scoring, and immunohistochemical quantification-were performed by investigators blinded to group allocation to minimize subjective bias.

A cranial defect model was established to assess the scaffolds' osteogenic capacity in vivo. Initially, anesthesia was induced using 3% w/v pentobarbital sodium. Circular 5 mm craniotomies were created bilaterally on the parietal bones, carefully avoiding the sagittal suture. The defects were filled with corresponding 5 mm scaffolds before skin closure. To minimize infection risk, penicillin (25,000 units/day) was administered for three days post-operation. The rats were maintained in a pathogen-free facility with regulated 12-h light/dark cycles.

At 4 and 12 weeks post-surgery, euthanasia was performed via pentobarbital sodium overdose. Harvested skull specimens were preserved in 4% paraformaldehyde at 4°C for 48 h before analysis.

### 5.15 Micro-computed tomography (micro-CT)

Micro-CT (SkyScan 1176) was used to assess the cranial defect sites at 4- and 12- weeks post-surgery. Three-dimensional bone reconstruction images were generated using CTvol software (version 2.0.0.4). Quantitative morphometric analysis was performed using CTAn to determine parameters including new bone volume (BV), new bone volume/total tissue volume (BV/TV), bone mineral density (BMD), trabecular separation (Tb.Sp), trabecular thickness (Tb.Th), and trabecular number (Tb.N).

### 5.16 Histology, immunohistochemistry (IHC) and immunofluorescence analysis

The collected specimens were processed through fixation, decalcification, gradual dehydration, and paraffin embedding. Sections of 3 μm thickness were prepared. Bone healing was qualitatively assessed using hematoxylin and eosin (H&E) staining and Masson's trichrome staining. For immunohistochemical (IHC) staining, anti-rat antibodies against ALP (BBL-0207, BASMEDTSCI), BMP-2 (BBL-0300, BASMEDTSCI), OCN (BA1076, BASMEDTSCI), and Runx2 (BA1093, BASMEDTSCI) were used to characterize new bone formation. Tissue sections were incubated with 3,3-diaminobenzidine (DAB, Vector Laboratories) for 4 min, followed by Mayer's hematoxylin counterstaining (Sigma-Aldrich) The slides were then examined under a microscope. For immunofluorescence staining targeting angiogenesis and macrophages, primary antibodies against CD31 (BA1019, BASMEDTSCI), CD86 (GB115630-50, Servicebio), and CD206 (GB123497-50, Servicebio) were employed. Fluorescence images were acquired using a Nikon confocal microscope. Quantitative analysis of CD206+/CD86+ cell ratios was performed using ImageJ (NIH).

### 5.17 Statistical analysis

All quantitative data were obtained from at least three independent biological replicates. Statistical analyses were performed using GraphPad Prism 10. Unpaired, two-tailed Student's t-test and One-way ANOVA were employed to assess data. The gathered data in this study were reported as the mean ± the standard deviation (SD). A p-value <0.05 was considered statistically significant.

## CRediT authorship contribution statement

**Yifan Zhang:** Conceptualization, Data curation, Formal analysis, Investigation, Methodology, Software, Validation, Visualization, Writing – original draft, Writing – review & editing. **Jie He:** Conceptualization, Data curation, Formal analysis, Investigation, Methodology, Software, Validation, Writing – review & editing. **Yuyang Zeng:** Investigation. **Yangyang Song:** Formal analysis, Investigation, Methodology, Software. **Zhengxing Wang:** Conceptualization, Resources, Validation. **Qian Wang:** Data curation, Formal analysis, Writing – review & editing. **Zhen You:** Project administration, Resources, Writing – review & editing. **Jiaming Sun:** Conceptualization, Funding acquisition, Methodology, Project administration, Resources, Supervision, Writing – review & editing.

## Declaration of competing interest

The authors declare the following financial interests/personal relationships which may be considered as potential competing interests: Jiaming Sun reports financial support was provided by National Natural Science Foundation of China. Zhen You reports financial support was provided by National Natural Science Foundation of China. Zhen You reports financial support was provided by Project of Natural Science Foundation of Sichuan Province. Qian Wang reports financial support was provided by General Natural Science Foundation of Sichuan. Zhenxing Wang reports was provided by Open Foundation of Hubei Key Laboratory of Regenerative Medicine and Multi-disciplinary Translational Research. Zhenxing Wang reports was provided by Interdiciplinary Research Program of Hust. If there are other authors, they declare that they have no known competing financial interests or personal relationships that could have appeared to influence the work reported in this paper.

## Data Availability

Data will be made available on request.
