# Supplementary material for: Multifunctional scaffold inspired by hepatocyte exosomes promotes bone regeneration by regulating osteogenic differentiation via PI3K/AKT pathway
Source: Mater Today Bio. 2026 Jun 20;39:103360. doi: 10.1016/j.mtbio.2026.103360 (PMC13316182; doi:10.1016/j.mtbio.2026.103360)
Supplement: Multimedia component 1 [file mmc1.docx]

**Supporting Information**

**Multifunctional Scaffold Inspired by Hepatocyte Exosomes Promotes Bone Regeneration by Regulating Osteogenic Differentiation via PI3K/AKT Pathway**

Yifan Zhang ^1#^, Jie He ^2#^, Yuyang Zeng ^3#^, Yangyang Song ^4^, Zhengxing Wang ^1,5^, Qian Wang ^6^*, Zhen You ^5, 6^*, and Jiaming Sun ^1^*

1. Department of Plastic Surgery, Union Hospital, Tongji Medical College, Huazhong University of Science and Technology, Wuhan, 430022, China.

2. Department of Neurosurgery, The Union Hospital of Tongji College, Huazhong University of Science and Technology, Wuhan 430022, P. R. China

3. Department of Dermatology, Tongji Hospital, Tongji Medical College, Huazhong

University of Science and Technology, Wuhan 430030, China.

4. Medical Genetics Center, Maternal and Child Health Hospital of Hubei Province, Wuhan, 430070, Hubei Province, China.

5. Hubei Key Laboratory of Regenerative Medicine and Multi-disciplinary Translational Research (Huazhong University of Science and Technology), Wuhan, Hubei 430022, China.

6. Department of Rehabilitation Medicine, Sichuan Provincial People’s Hospital, School of Medicine, University of Electronic Science and Technology of China, Chengdu 610065, Sichuan, China; Rehabilitation Medicine Center, West China Hospital, Sichuan University, Chengdu 610065, Sichuan, China

7. Division of Biliary Surgery, Department of General Surgery, West China Hospital, Sichuan University, Chengdu 610041, Sichuan, China; Research Center for Biliary Diseases, West China Hospital, Sichuan University, Chengdu 610041, Sichuan, China

^#^. These authors contributed equally to this work.

*. Authors to whom any correspondence should be addressed

E-mail: Jiaming Sun (sunjm1592@sina.com),

Zhen You ([youzhen@wchscu.cn](mailto:youzhen@wchscu.cn))

Qian Wang (wangqianwind@163.com)


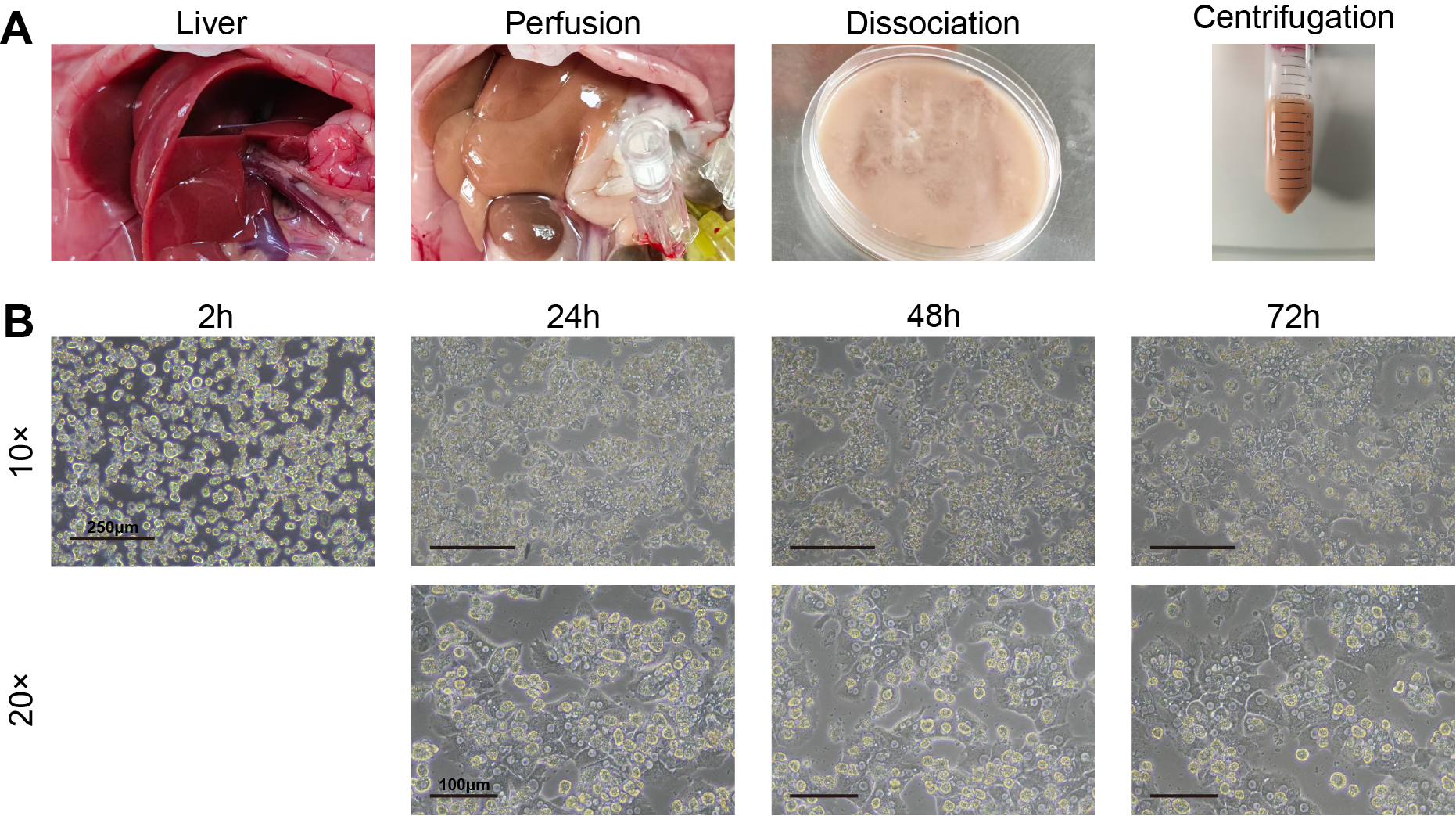


**Figure S1. A.** Schematic illustration of the different steps involved in hepatocyte isolation. **B.** Light microscopy images of hepatocytes at different culture time points.


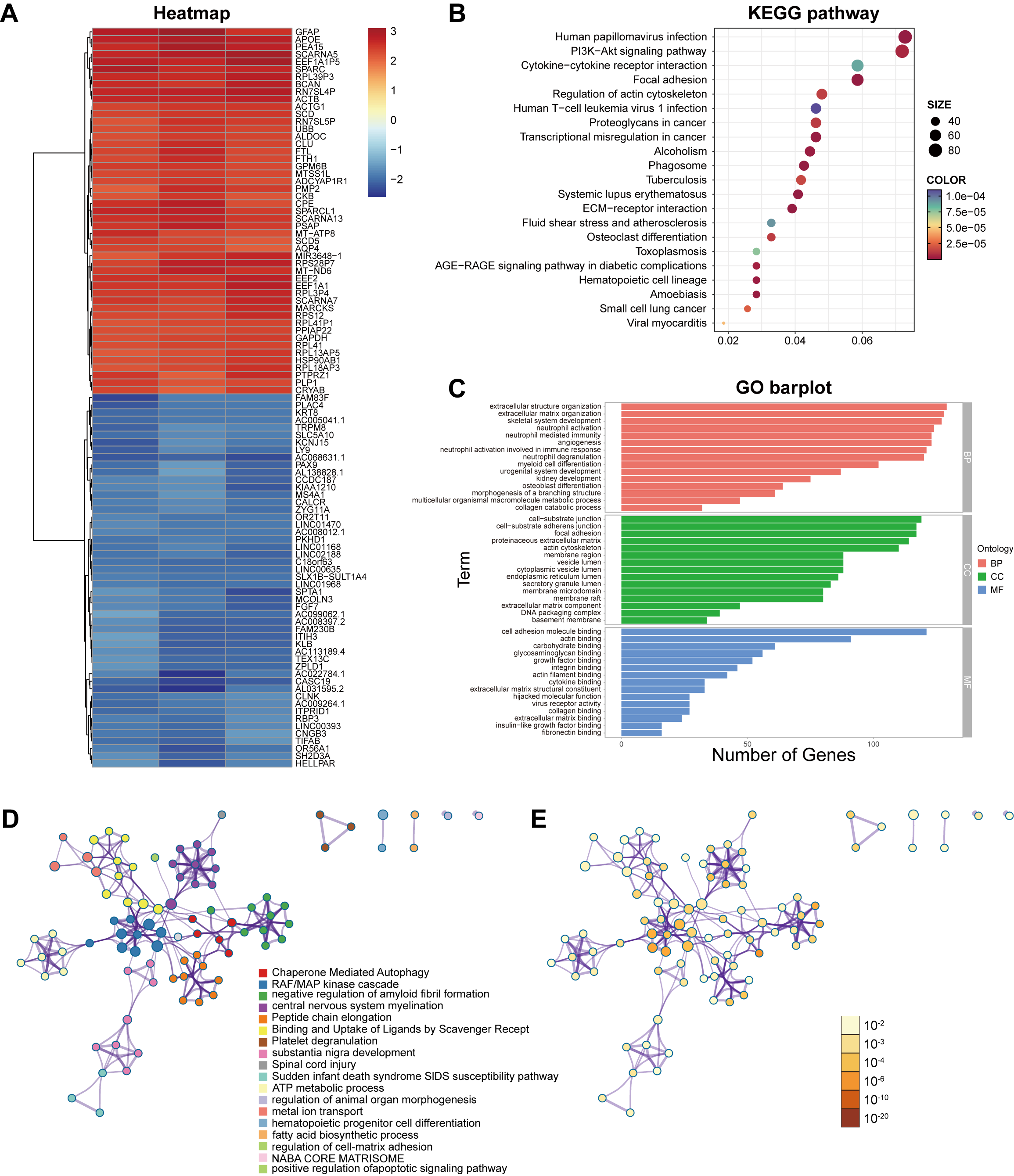


**Figure S2.** RNA-Sequencing of h-EXOs. **A.** Heatmap of h-EXOs sequencing analysis. **B-C.** KEGG pathway enrichment and GO analysis reveal pathways closely associated with various biological processes. **D-E.** Protein-Protein Interaction (PPI) Network


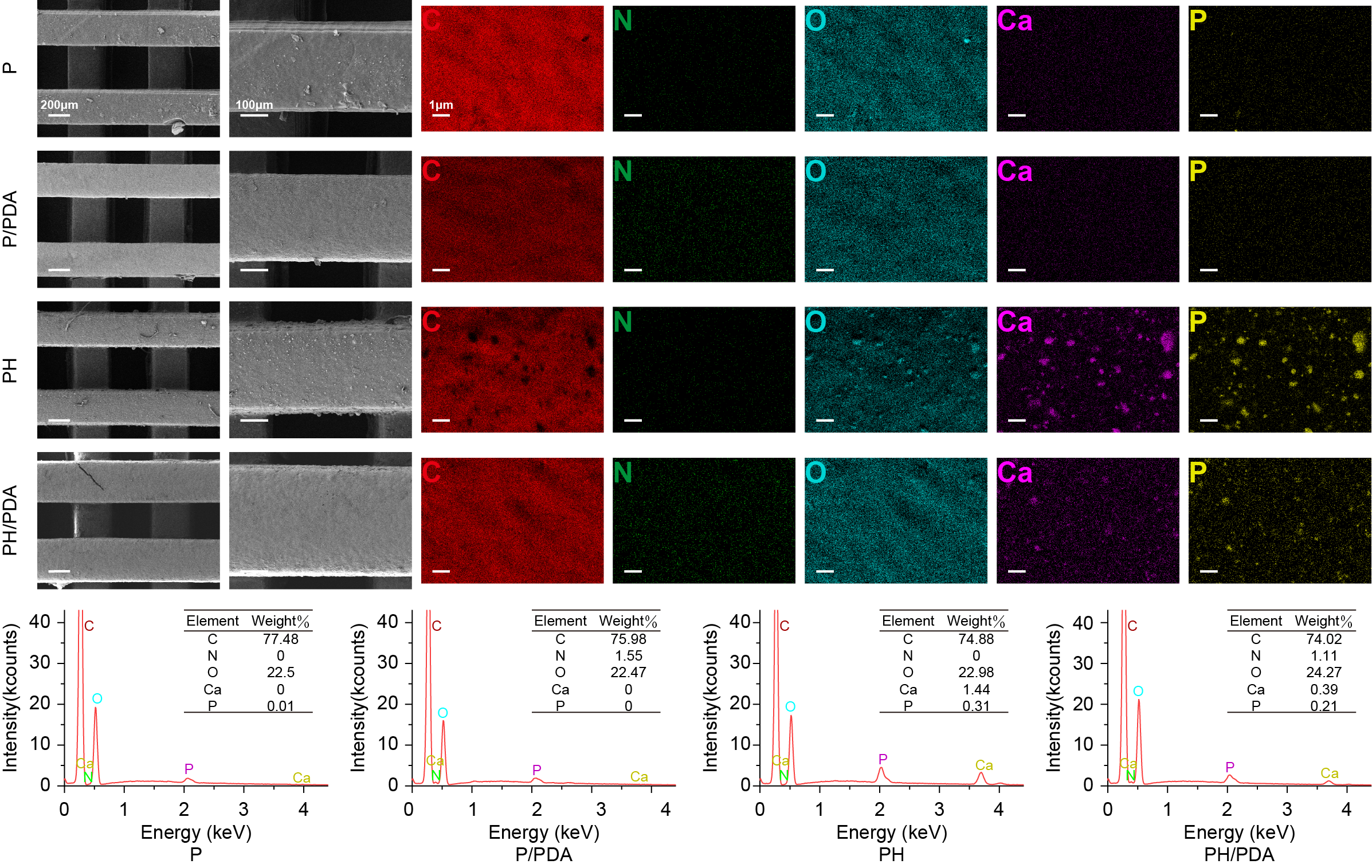


**Figure S3.** Low-magnification SEM images and EDS-mapping analyse revealing the structural characteristics of different scaffold.


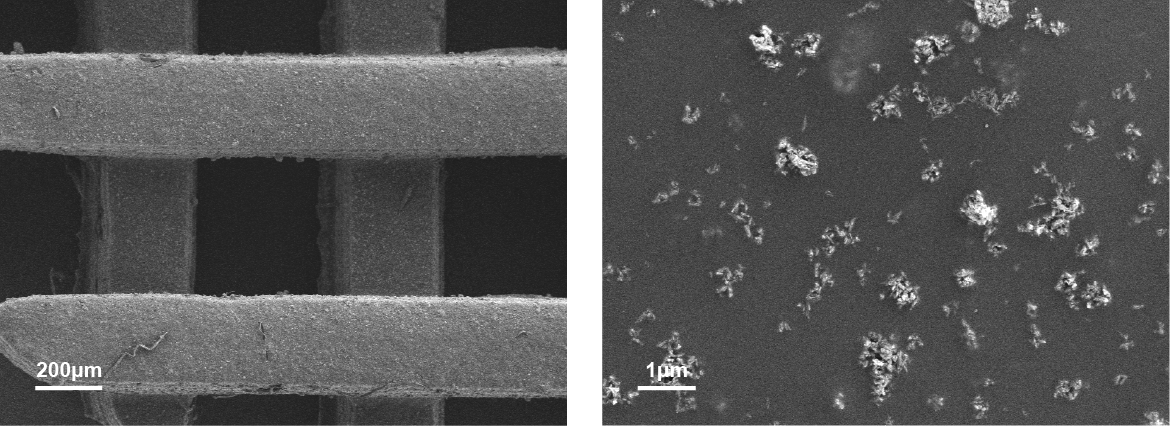


**Figure S4.** Low-magnification SEM images of PH /h-EXOs.


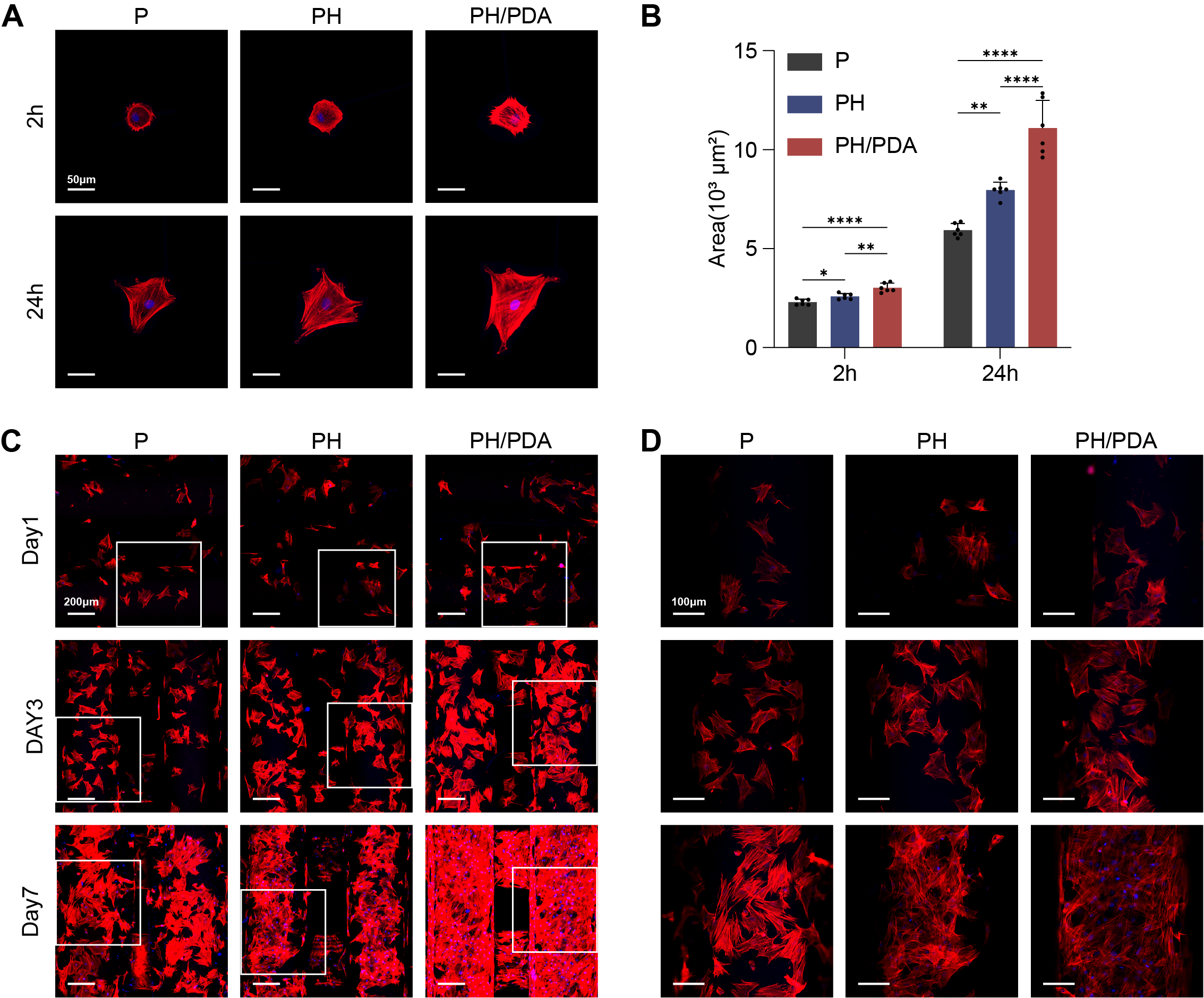


**Figure S5.** **A.** Morphology of BMSCs seeded on P, PH, and PH/PDA scaffolds after 2 and 24 hours. **B.** Cytomorphometry analysis based on images. (n = 6) **C.** Comparison of BMSC morphology and cell number after culture on different scaffolds for 1, 3, and 7 days. Red and blue indicate actin filaments and cell nuclei, respectively. **D.** Magnified view of BMSCs on the scaffold. *p < 0.05, **p < 0.01, **** p < 0.0001


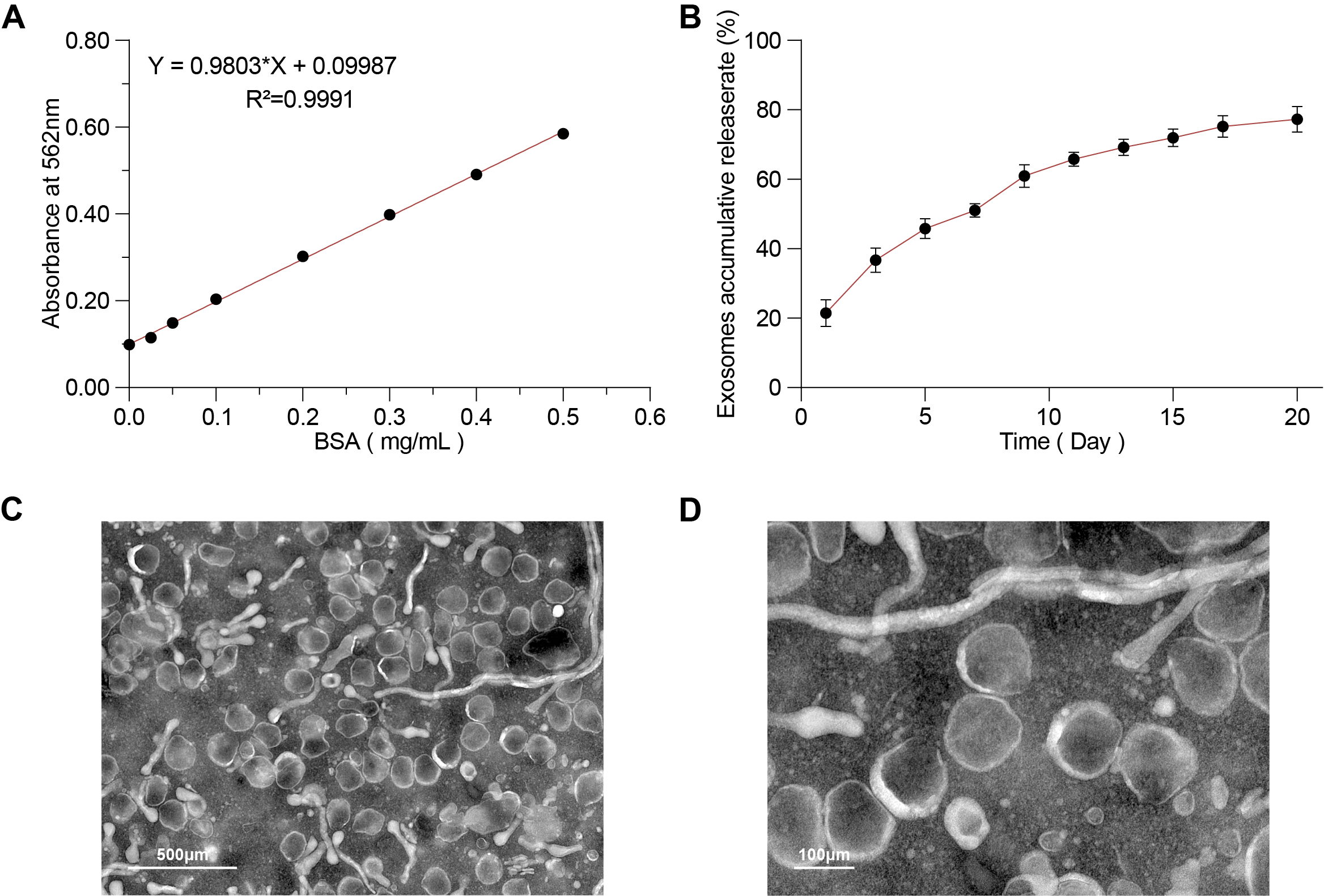


**Figure S6**. **A.** BCA protein content standard curve. **B.** The release performance of the h-EXOs. **C-D.** Observation of the morphology of h-EXOs. released from the scaffold at day 20 under TEM.


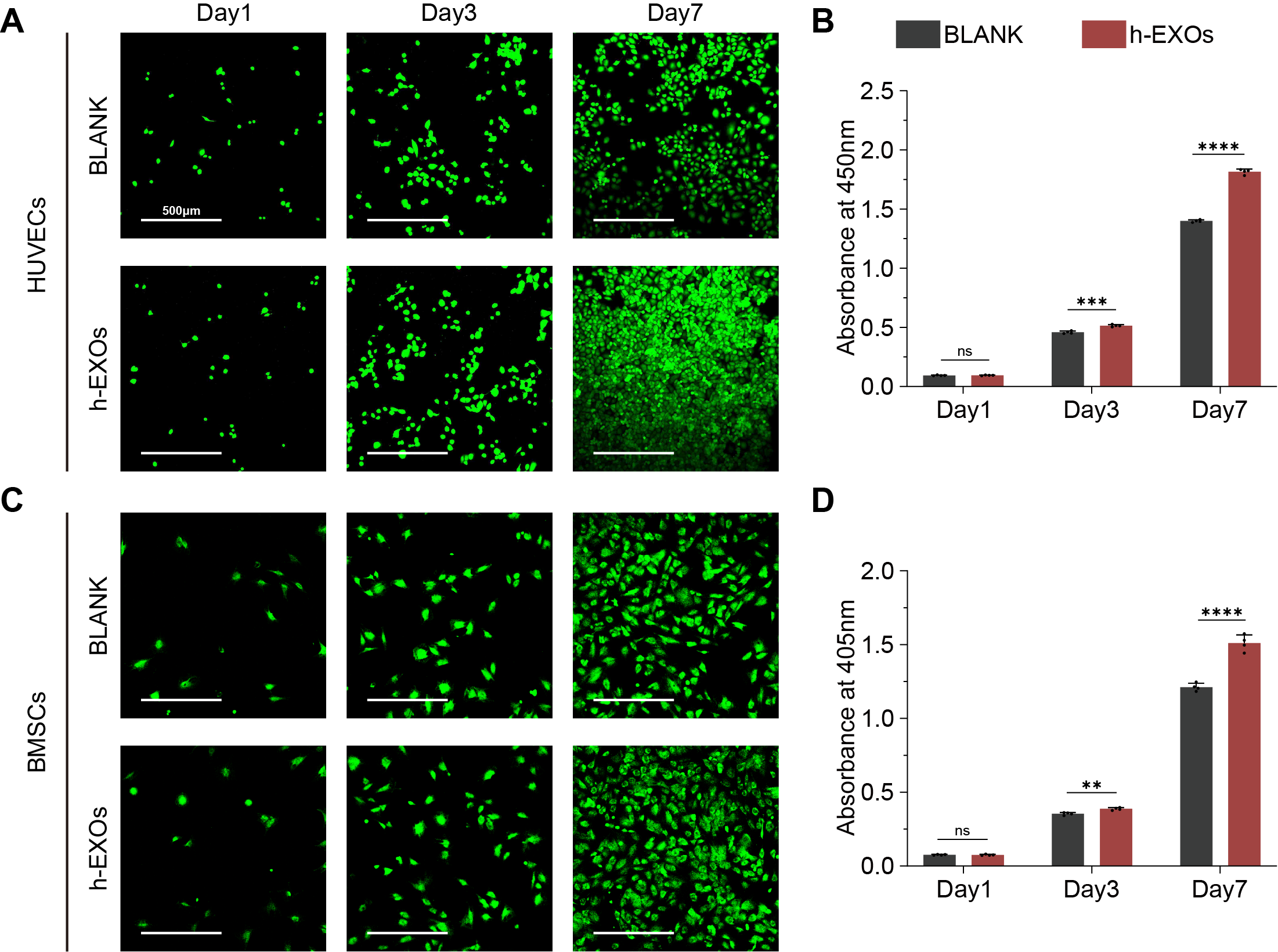


**Figure S7.** **A-B.** HUVECs were cultured with late-stage released h-EXOs, and Calcein AM/PI staining and CCK-8 assays were performed on days 1, 3, and 7. (n = 4) **C-D.** A-B. HUVECs were cultured with late-stage released h-EXOs, and Calcein AM/PI staining and CCK-8 assays were performed on days 1, 3, and 7. (n = 4) **p < 0.01, *** p < 0.001, **** p < 0.0001.


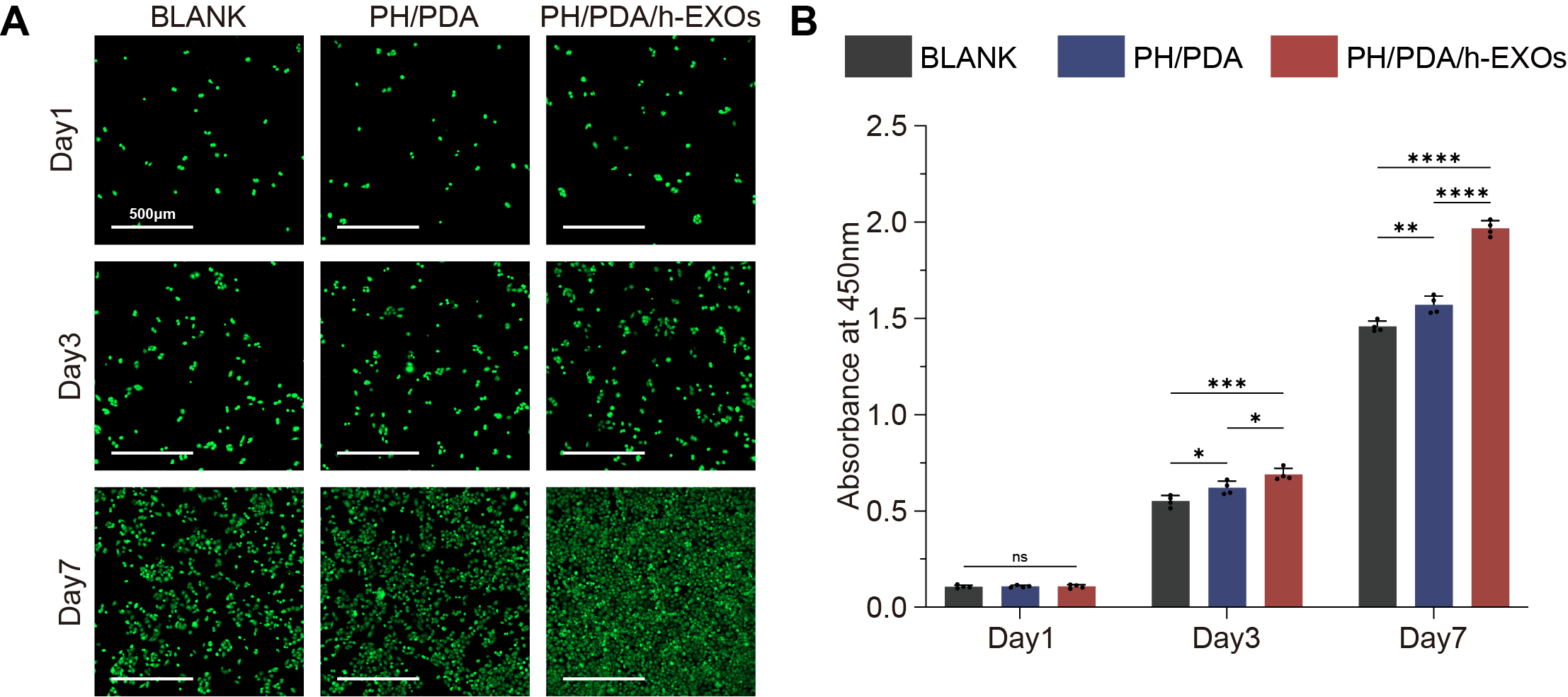


**Figure S8.** **A-B.** Calcein-AM/PI staining and CCK-8 assay of BMSCs seeded on scaffolds at days 1, 3, and 7. (n = 4) *p < 0.05, **p < 0.01, *** p < 0.001, **** p < 0.0001


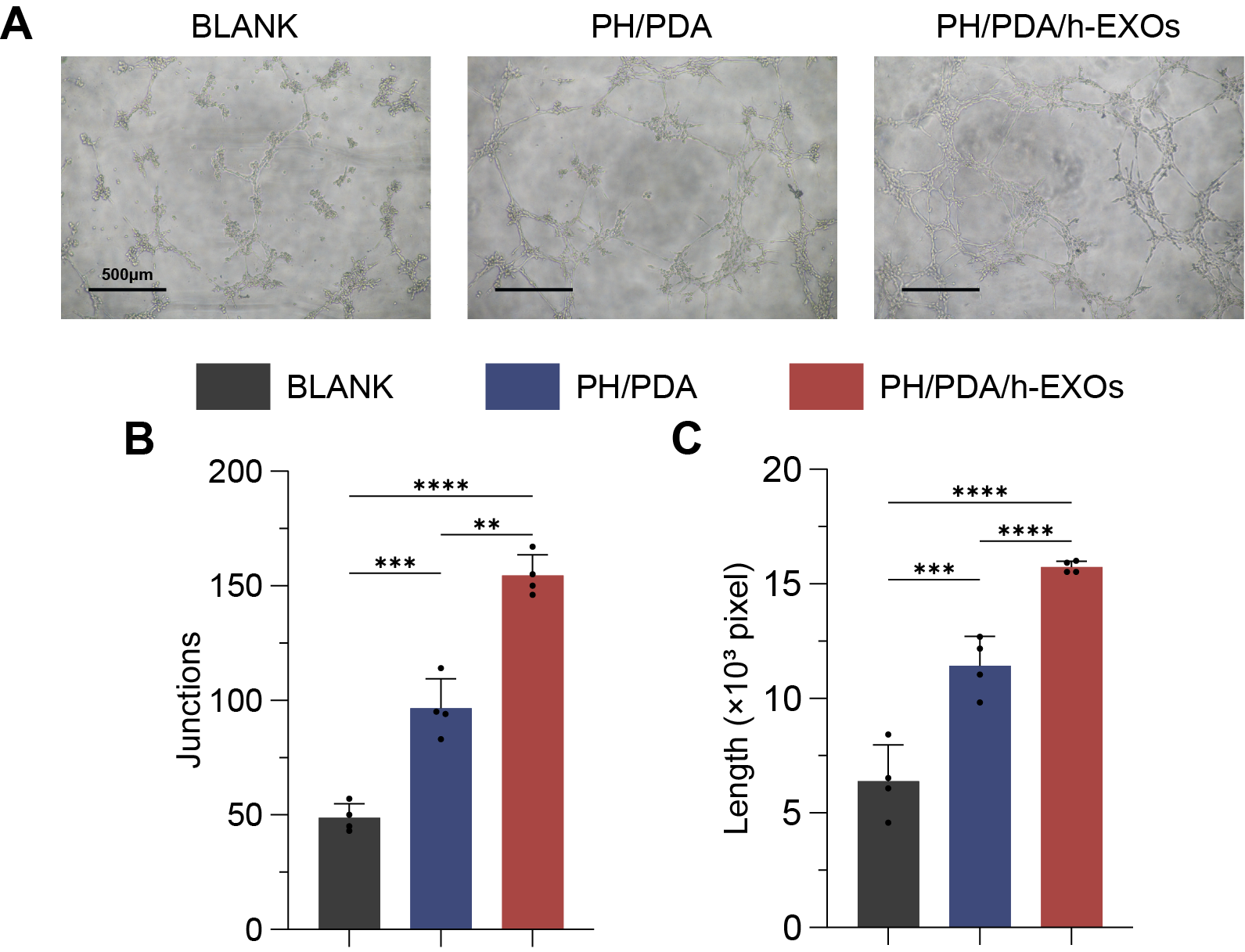


**Figure S9. A.** Representative images of tube formation in different groups.Scale bar = 500 μm. **B-C.** Statistical analysis of number of junctions (**B**) and total branching length (**C**) representing tube formation ability. (n=4) **p < 0.01, ***p < 0.001, **** p < 0.0001


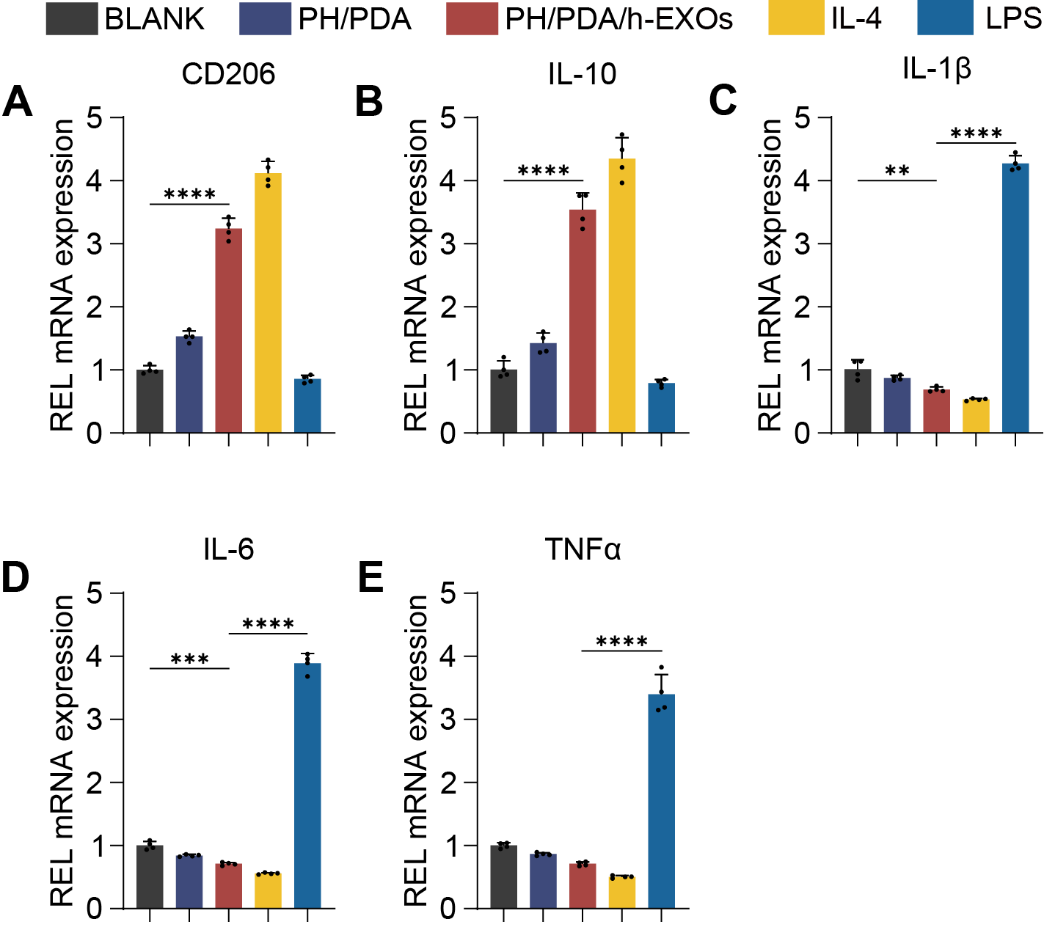


**Figure S10.** **A-E.** The levels of CD206, IL-10, IL-1β, IL-6 and TNF-α mRNA expression were measured by qRT-PCR under the influence of different scaffolds. (n = 4) **p < 0.01, *** p < 0.001, **** p < 0.0001


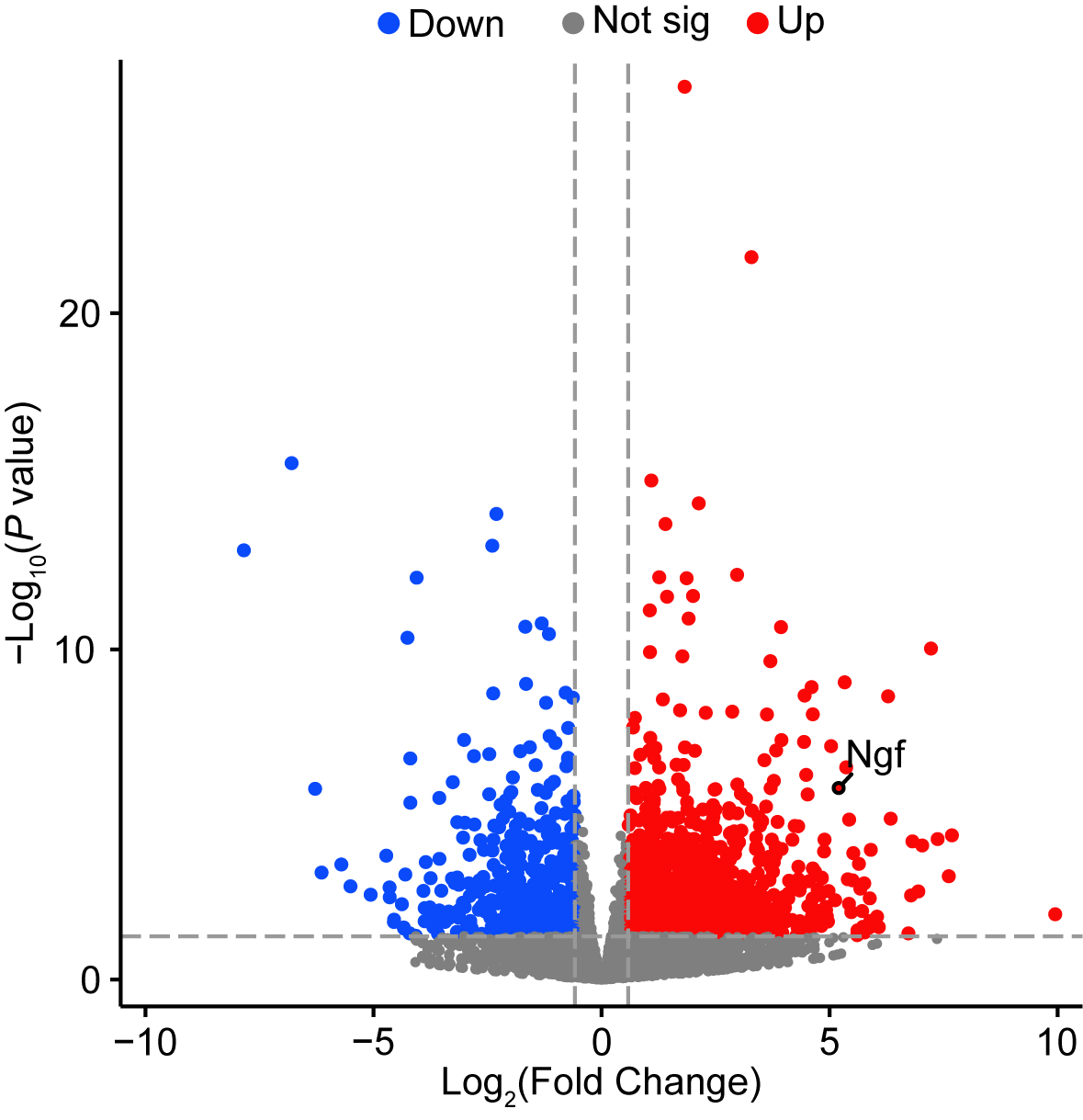


**Figure S11.** Volcano map of differentially expressed proteins.


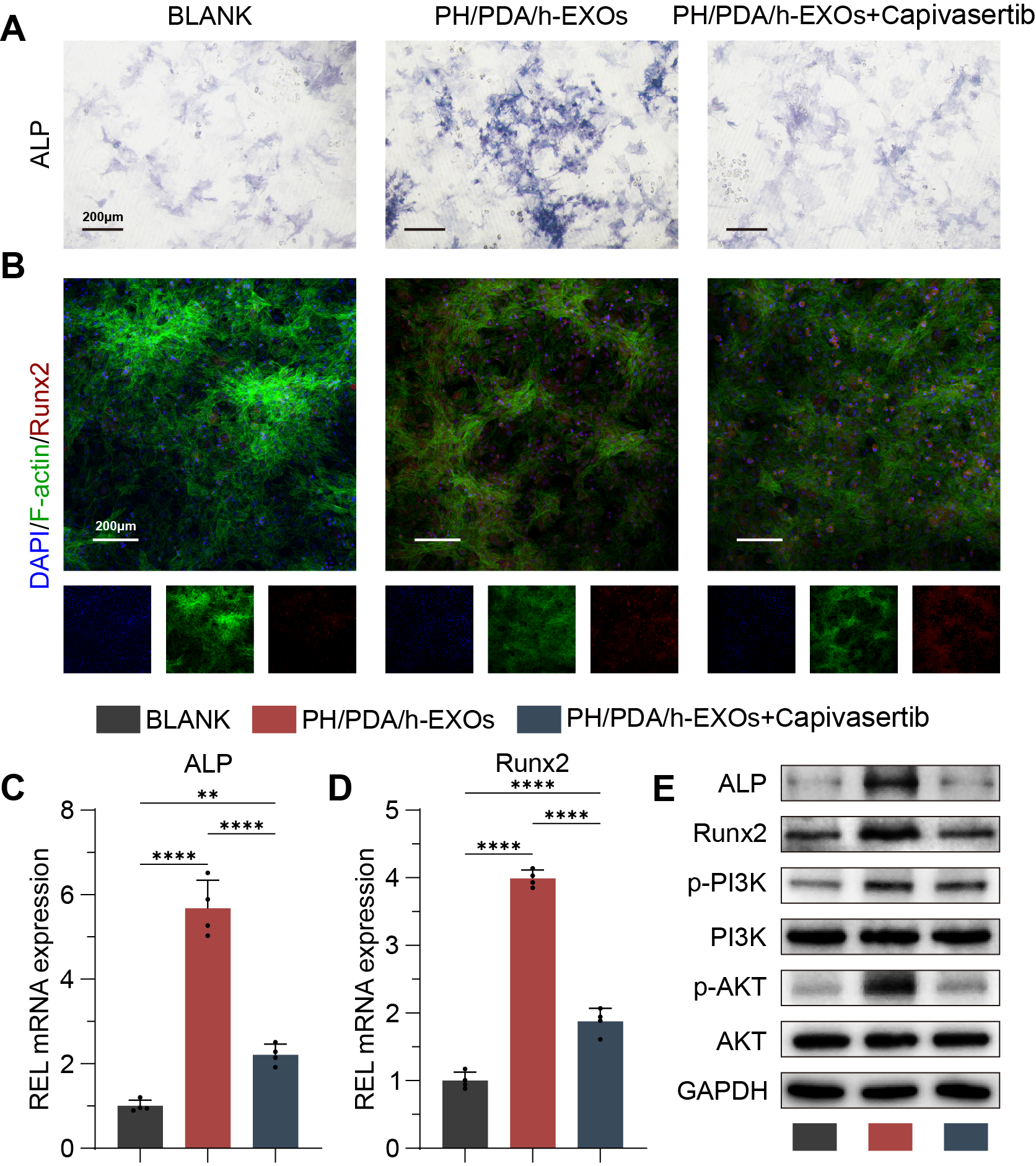


**Figure S12.** **A.** ALP staining images after osteogenic induction with different scaffolds on days 7. **B.** IF staining of osteogenic markers Runx2 in BMSCs induced by different scaffolds. **C.** Osteogenic markers ALP assessed by qRT-PCR at days 7. (n = 4) **D.** Osteogenic markers Runx2 assessed by qRT-PCR at days 7. (n = 4) **E.** Expression of key proteins in BMSCs cultured on different scaffolds for 7 days, analyzed by WB. **p < 0.01, **** p < 0.0001


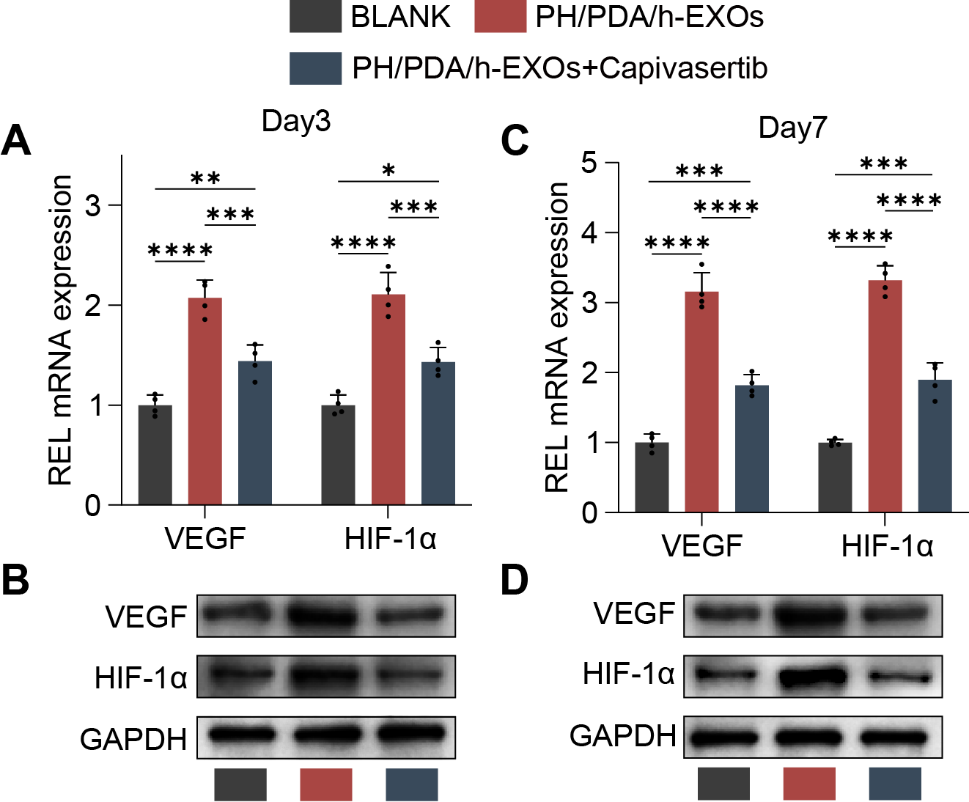


**Figure S13.** A-B. Expression levels of VEGF and HIF-1α genes assessed by qRT-PCR and WB on days 3 of co-culture. (n = 4) C-D. Expression levels of VEGF and HIF-1α genes assessed by qRT-PCR and WB on days 7 of co-culture. (n = 4) *p < 0.05, **p < 0.01, *** p < 0.001, **** p < 0.0001.

**
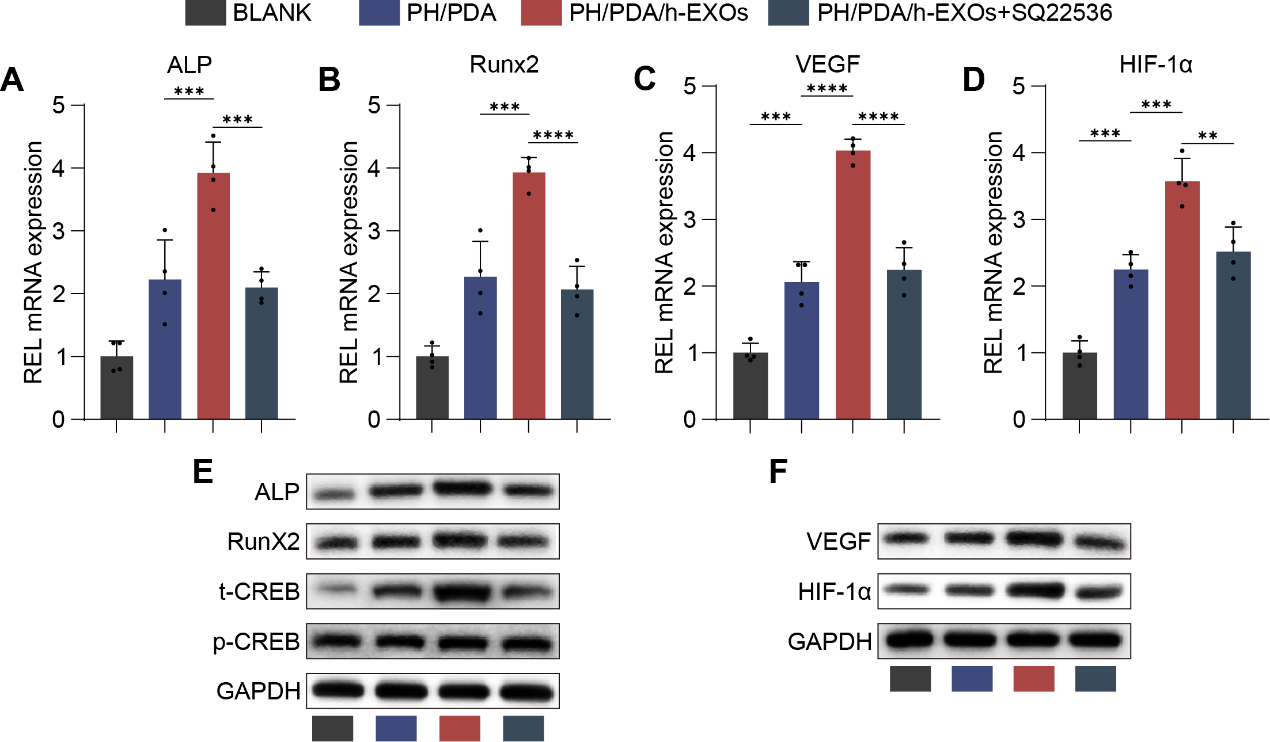
**

**Figure S14.** **A-B.** Osteogenic markers ALP and Runx2 assessed by qRT-PCR at days 7. (n = 4) **C-D.** Expression levels of VEGF and HIF-1α assessed by qRT-PCR at days 3. (n = 4) **E-F.** Expression of key proteins in BMSCs and HUVECs cultured on different scaffolds, analyzed by WB. **p < 0.01, *** p < 0.001, **** p < 0.0001


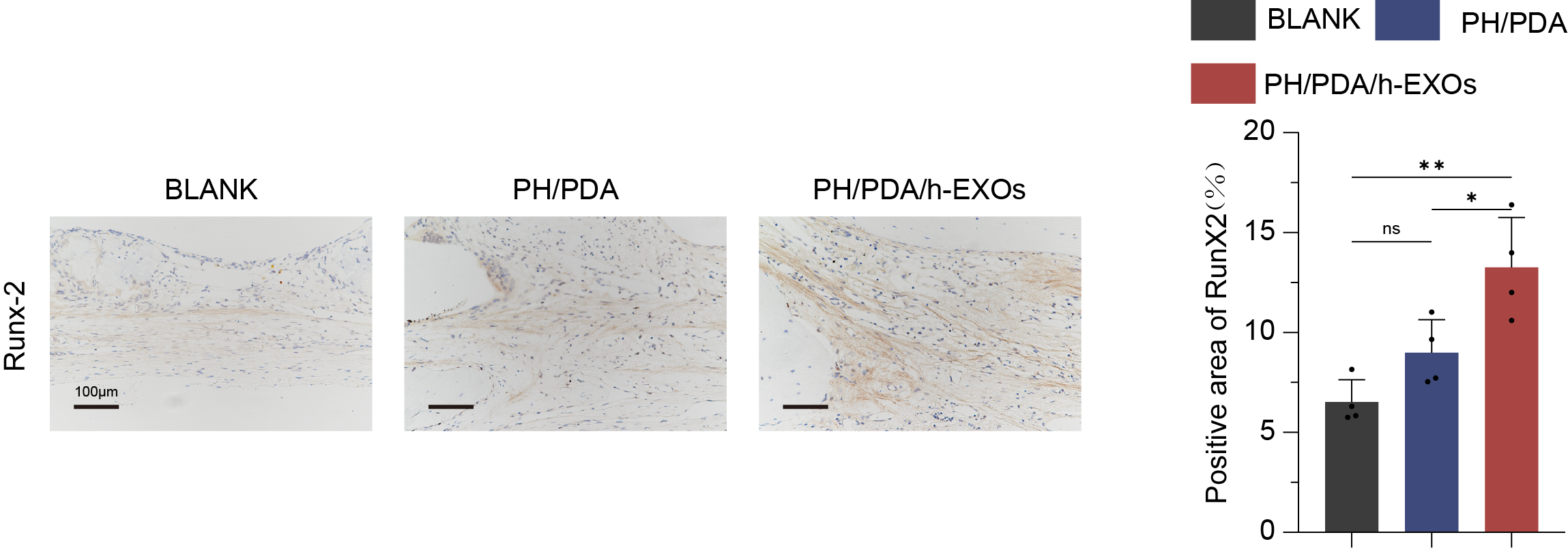


**Figure S15.** Representative images and quantitative analysis of immunohistochemical staining for Runx-2. (n = 4) *p < 0.05, **p < 0.01.


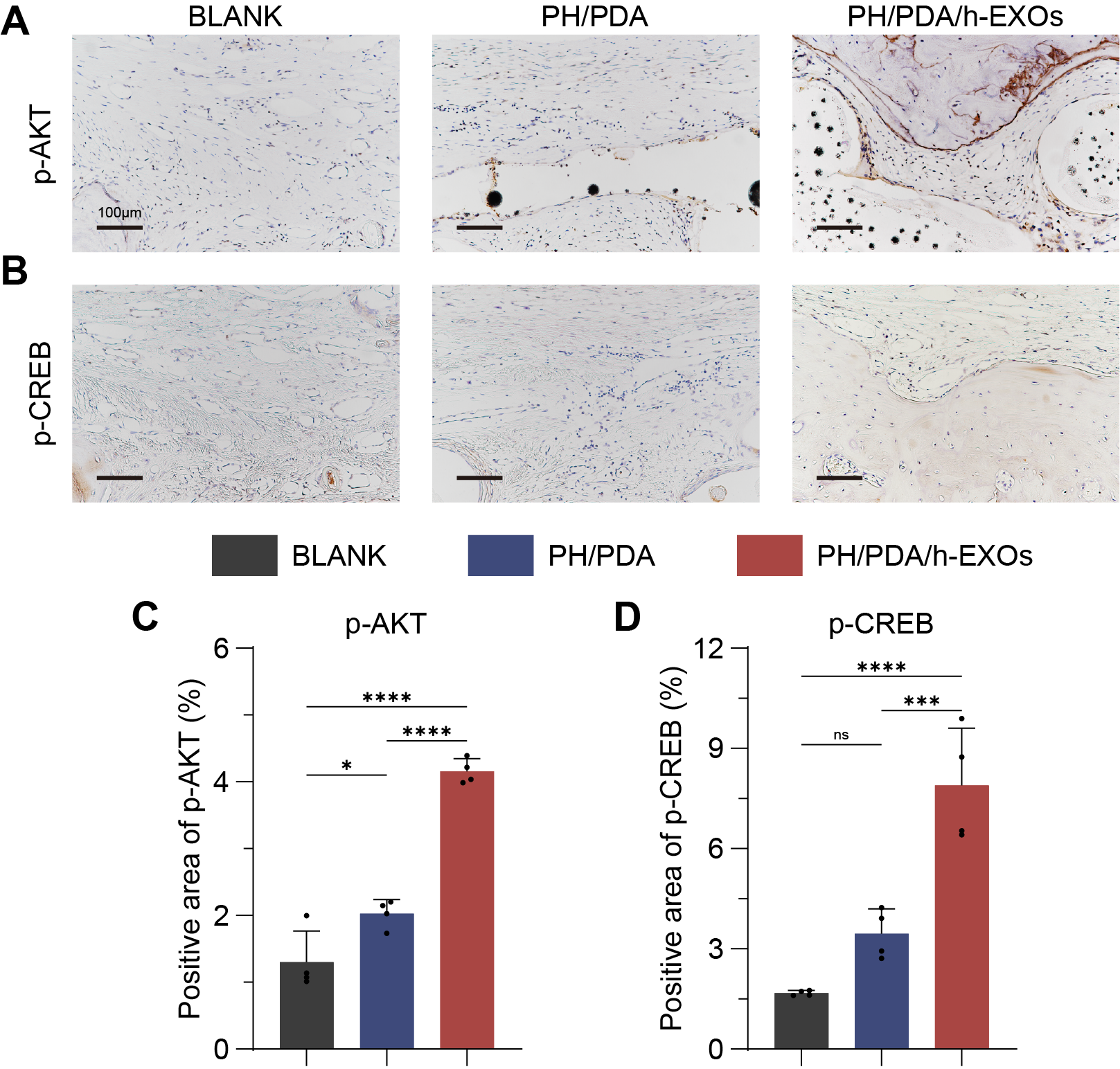


**Figure S16.** **A-B.** Representative images of immunohistochemical staining for p-AKT and p-CREB. **C-D.** Quantitative analysis of immunohistochemical staining for p-AKT and p-CREB. (n = 4) *p < 0.05, *** p < 0.001, **** p < 0.0001.


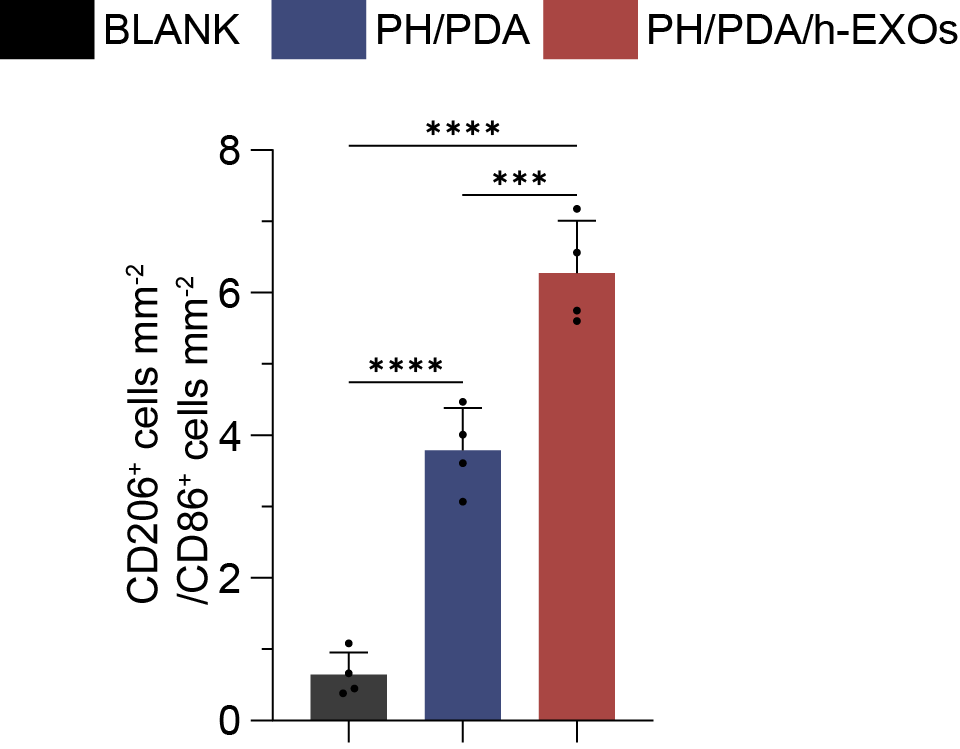


**Figure S17.** Quantitative analysis of CD206/86 in each field. (n = 4) *** p < 0.001, **** p < 0.0001

| Gene | Forward Primer | Reverse Primer |
| --- | --- | --- |
| GAPDH | AGGTCGGTGTGAACGGATTTG | TGTAGACCATGTAGTTGAGGTCA |
| ALP | CCAACTCTTTTGTGCCAGAGA | GGCTACATTGGTGTTGAGCTTTT |
| Runx2 | TTCAACGATCTGAGATTTGTGGG | GGATGAGGAATGCGCCCTA |
| BMP-2 | ACCCGCTGTCTTCTAGCGT | TTTCAGGCCGAACATGCTGAG |
| COLⅠA1 | GAGGGCCAAGACGAAGACATC | CAGATCACGTCATCGCACAAC |
| OPN | ATCTCACCATTCGGATGAGTCT | TGTAGGGACGATTGGAGTGAAA |
| OCN | CTGACCTCACAGATCCCAAGC | TGGTCTGATAGCTCGTCACAAG |
| HIF-1α | GATGACGGCGACATGGTTTAC | CTCACTGGGCCATTTCTGTGT |
| VEGF | GCACATAGAGAGAATGAGCTTCC | CTCCGCTCTGAACAAGGCT |
| ANG | CTGGGCGTTTTGTTGTTGGTC | GGTTTGGCATCATAGTGCTGG |
| vWF | CCGATGCAGCCTTTTCGGA | TCCCCAAGATACACGGAGAGG |
| CD31 | AACAGTGTTGACATGAAGAGCC | TGTAAAACAGCACGTCATCCTT |

**Table 1.** Primer sequences for qRT-PCR analysis


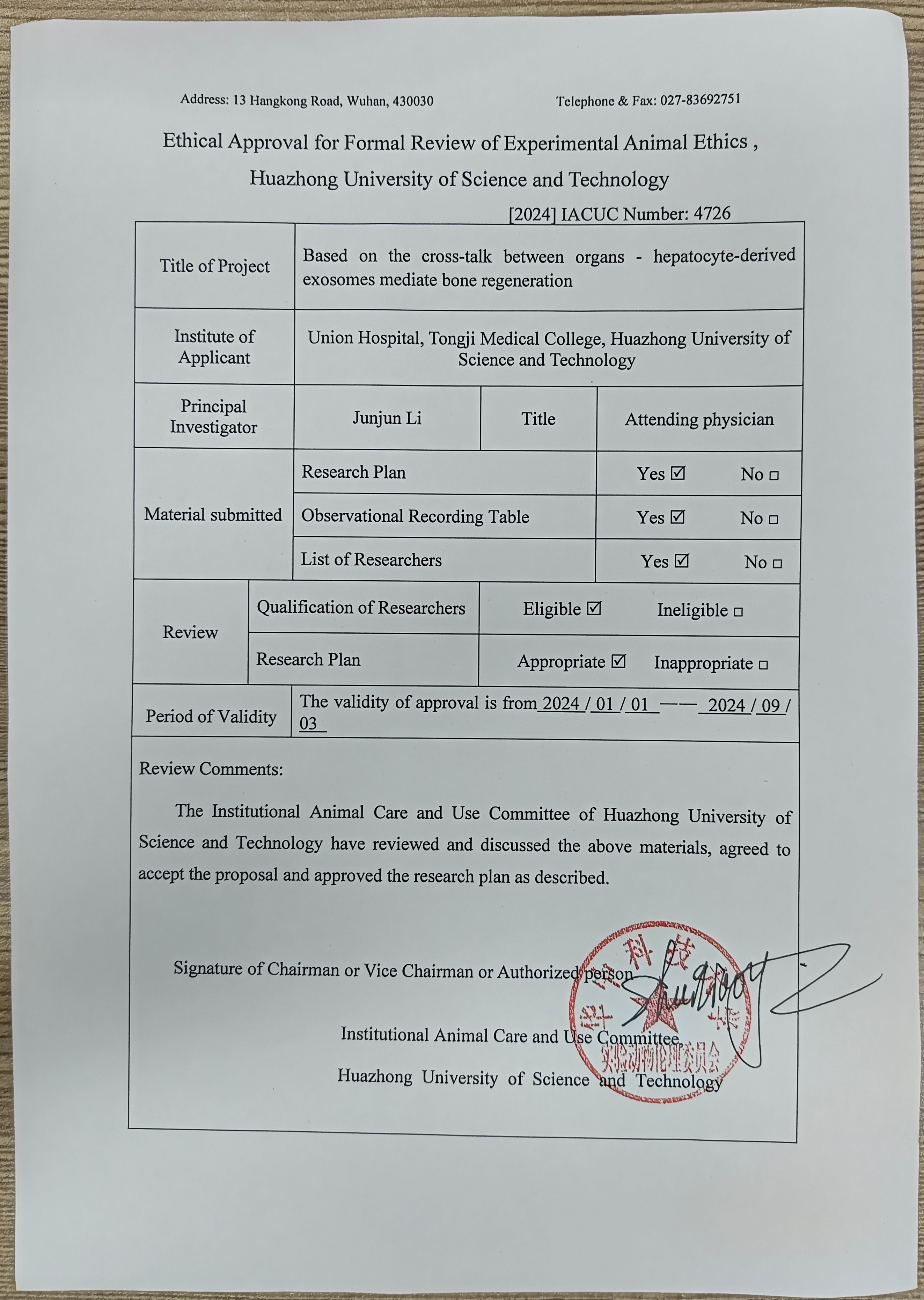


**Document1.** Ethical approval document
