# Supplementary material for: Multifunctional scaffold inspired by hepatocyte exosomes promotes bone regeneration by regulating osteogenic differentiation via PI3K/AKT pathway
Source: Mater Today Bio. 2026 Jun 20;39:103360. doi: 10.1016/j.mtbio.2026.103360 (PMC13316182; doi:10.1016/j.mtbio.2026.103360)
Supplement: Multimedia component 2 [file mmc2.doc]

| Random Number Table | | | | | | | | | | | | | | |
| --- | --- | --- | --- | --- | --- | --- | --- | --- | --- | --- | --- | --- | --- | --- |
| 91 | 77 | 63 | 39 | 3 | 9 | 88 | 62 | 97 | 26 | 96 | 5 | 36 | 75 | 1 |
| 14 | 47 | 47 | 95 | 62 | 60 | 71 | 74 | 50 | 6 | 31 | 36 | 17 | 31 | 72 |
| 14 | 53 | 29 | 35 | 12 | 67 | 86 | 35 | 91 | 86 | 3 | 60 | 52 | 33 | 31 |
| 17 | 74 | 39 | 60 | 23 | 71 | 14 | 85 | 24 | 32 | 90 | 79 | 44 | 71 | 11 |
| 52 | 15 | 76 | 82 | 92 | 88 | 60 | 4 | 49 | 20 | 39 | 34 | 53 | 71 | 100 |
| 59 | 78 | 58 | 91 | 17 | 86 | 25 | 71 | 93 | 83 | 66 | 34 | 49 | 68 | 31 |
| 39 | 69 | 37 | 73 | 65 | 30 | 88 | 64 | 77 | 60 | 86 | 33 | 77 | 83 | 83 |
| 70 | 73 | 82 | 42 | 77 | 30 | 28 | 16 | 97 | 60 | 77 | 28 | 97 | 1 | 34 |
| 22 | 56 | 34 | 75 | 71 | 13 | 87 | 80 | 83 | 97 | 80 | 61 | 79 | 20 | 36 |
| 5 | 68 | 38 | 88 | 35 | 64 | 17 | 25 | 83 | 50 | 88 | 29 | 62 | 92 | 41 |

**Table 2.** Random number table generated using Excel.

| Animal Number | Random Number | Reorder | Group | |
| --- | --- | --- | --- | --- |
| 1 | 59 | 7 | PH/PDA |  |
| 2 | 78 | 11 | PH/PDA/h-EXOs |  |
| 3 | 58 | 6 | PH/PDA |  |
| 4 | 91 | 14 | PH/PDA/h-EXOs |  |
| 5 | 17 | 1 | Blank |  |
| 6 | 86 | 13 | PH/PDA/h-EXOs |  |
| 7 | 25 | 2 | Blank |  |
| 8 | 71 | 10 | PH/PDA |  |
| 9 | 93 | 15 | PH/PDA/h-EXOs |  |
| 10 | 83 | 12 | PH/PDA/h-EXOs |  |
| 11 | 66 | 8 | PH/PDA |  |
| 12 | 34 | 4 | Blank |  |
| 13 | 49 | 5 | Blank |  |
| 14 | 68 | 9 | PH/PDA |  |
| 15 | 31 | 3 | Blank |  |

**Table 3**. Starting from the first column of the sixth row, the random numbers were ranked (with duplicate values excluded) and used for animal group allocation.
